# SseK1 and SseK3 Type III Secretion System Effectors Inhibit NF-κB Signaling and Necroptotic Cell Death in Salmonella-Infected Macrophages

**DOI:** 10.1128/IAI.00010-17

**Published:** 2017-02-23

**Authors:** Regina A. Günster, Sophie A. Matthews, David W. Holden, Teresa L. M. Thurston

**Affiliations:** Section of Microbiology, Medical Research Council Centre for Molecular Bacteriology and Infection, Imperial College London, London, United Kingdom; University of California, Davis

**Keywords:** NF-κB signaling, Salmonella, cell death, necroptosis

## Abstract

Within host cells such as macrophages, Salmonella enterica translocates virulence (effector) proteins across its vacuolar membrane via the SPI-2 type III secretion system. Previously, it was shown that when expressed ectopically, the effectors SseK1 and SseK3 inhibit tumor necrosis factor alpha (TNF-α)-induced NF-κB activation. In this study, we show that ectopically expressed SseK1, SseK2, and SseK3 suppress TNF-α-induced, but not Toll-like receptor 4- or interleukin-induced, NF-κB activation. Inhibition required a DXD motif in SseK1 and SseK3, which is essential for the transfer of *N*-acetylglucosamine to arginine residues (arginine-GlcNAcylation). During macrophage infection, SseK1 and SseK3 inhibited NF-κB activity in an additive manner. SseK3-mediated inhibition of NF-κB activation did not require the only known host-binding partner of this effector, the E3-ubiquitin ligase TRIM32. SseK proteins also inhibited TNF-α-induced cell death during macrophage infection. Despite SseK1 and SseK3 inhibiting TNF-α-induced apoptosis upon ectopic expression in HeLa cells, the percentage of infected macrophages undergoing apoptosis was SseK independent. Instead, SseK proteins inhibited necroptotic cell death during macrophage infection. SseK1 and SseK3 caused GlcNAcylation of different proteins in infected macrophages, suggesting that these effectors have distinct substrate specificities. Indeed, SseK1 caused the GlcNAcylation of the death domain-containing proteins FADD and TRADD, whereas SseK3 expression resulted in weak GlcNAcylation of TRADD but not FADD. Additional, as-yet-unidentified substrates are likely to explain the additive phenotype of a Salmonella strain lacking both SseK1 and SseK3.

## INTRODUCTION

Salmonella enterica is a facultative intracellular pathogen whose serovars cause a range of diseases in mammals, diseases associated with both localized and systemic infections. Numerous virulence proteins are important for the colonization and infection of its hosts (1). One important component of Salmonella virulence is the Salmonella pathogenicity island 2 (SPI-2)-encoded type III secretion system (T3SS), which enables the bacterium to translocate virulence (effector) proteins across the Salmonella-containing vacuole (SCV) into the host cell. To date, approximately 30 effectors that are translocated via this secretion apparatus have been identified (2). Several of these have been proposed or shown to interfere with the activity of key signaling pathways required for the induction of an innate immune response. These effectors include GogB (3), SspH1 (4), SpvC (5), SpvD (6), and GtgA (7).

A major innate immune signaling pathway targeted by many bacterial pathogens (including Salmonella) is the nuclear factor κB (NF-κB) pathway (8–12). This pathway is critical to drive the innate and adaptive immune responses, including the induction of proinflammatory cytokines (13) and the control of cell survival (14). Various bacterial components and host cell cytokines, such as tumor necrosis factor alpha (TNF-α), activate this conserved signaling pathway of phosphorylation and ubiquitination events that culminates in the translocation of the NF-κB transcription factors into the host cell nucleus (15). TNF-α binds to the TNF receptor 1 (TNFR1), which subsequently initiates the NF-κB pathway via death domain-containing proteins (16). In addition, engagement of TNFR1 by TNF-α initiates the formation of the death-inducing signaling complex (DISC), consisting of the FAS-associated death domain protein (FADD), TNFR1-associated death domain protein (TRADD), and pro-caspase-8, ultimately resulting in apoptotic cell death (17). Alternatively, if the levels of caspase-8 activity are low, then RIPK1 (rip interacting protein kinase 1) and RIPK3 associate, auto- and transphosphorylate each other, and form a complex that phosphorylates MLKL (mixed-lineage kinase domain-like), which is required for the induction of necroptosis (18–20).

The enteropathogenic and enterohemorrhagic Escherichia coli T3SS effector NleB inhibits death domain-containing proteins, including FADD and TRADD, leading to reduced NF-κB pathway activation and impaired caspase-8-dependent host cell death during infection (21, 22). NleB is an *N*-acetylglucosamine (GlcNAc) transferase (21–23) that, unlike host enzymes, catalyzes a probably irreversible modification of arginine residues within the death domains of target proteins (21). Salmonella enterica serovar Typhimurium encodes three SPI-2 T3SS effectors with sequence similarity to NleB (24): SseK1, SseK2, and SseK3. These effectors contain the essential divalent cation and/or sugar-coordinating DXD motif that is required for enzymatic function of glycosyltransferases of the GT-A family (25). Despite their similarity to NleB, the SseK family remains poorly characterized. Following expression after transfection, SseK1 inhibits the NF-κB pathway, and like NleB, GlcNAcylates TRADD (21). Data reported by Yang et al. (26) suggested that SseK3 also inhibits the NF-κB pathway following transfection; however, direct evidence for SseK-mediated NF-κB inhibition during Salmonella infection is lacking.

Here, we report that both SseK1 and SseK3 inhibit Salmonella-induced NF-κB activation and necroptotic cell death during infection in macrophages. Transfection-based experiments to delineate the selectivity of inhibition reveal specificity of all three SseK proteins toward TNF-α-driven NF-κB activation, rather than inhibition of signaling that is common to different stimuli. In addition, SseK proteins inhibit TNF-α-induced cell death during macrophage infection. SseK3-mediated inhibition of NF-κB activation does not require the E3-ubiquitin ligase tripartite motif-containing protein 32 (TRIM32), its only known binding partner (26). During infection of macrophages, expression of SseK1 and SseK3 leads to clearly distinct profiles of arginine-GlcNAcylated proteins. This, together with their differential abilities to modify TRADD and FADD, reveals that the SseK effectors have distinct host cell targets during Salmonella infection.

## RESULTS

### Translocation and intracellular localization of SseK effectors in macrophages.

**FIG 1:**
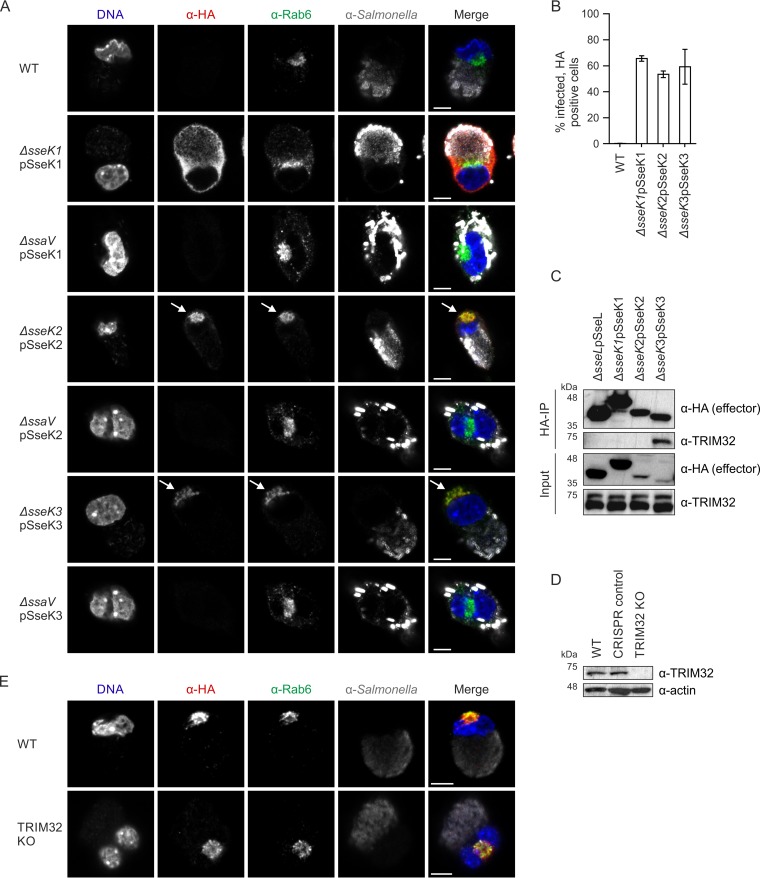
SseK effector translocation and localization in macrophages. (A) Representative images by confocal immunofluorescence microscopy of RAW 264.7 macrophages infected with wild-type (WT) or the indicated mutant Salmonella strains expressing HA-tagged SseK effectors at 16 hpu: Salmonella (anti-CSA-1 [α-CSA-1], gray), effectors (α-HA, red), Golgi network (α-Rab6, green), DNA (DAPI, blue). Bar, 5 μm. Effector colocalization with the Golgi network is highlighted with arrows. (B) Percentage of infected cells with translocated HA-tagged SseK effectors, quantified by immunofluorescence microscopy at 16 hpu. A total of at least 600 infected cells were counted in three independent experiments. Values shown are mean results ± SEM. (C) RAW 264.7 macrophages were infected for 16 h with the indicated Salmonella strains, lysed, and proteins were immunoprecipitated (IP) with antibody α-HA-agarose. Samples were analyzed by SDS-PAGE and immunoblotted for effectors (α-HA) and TRIM32 (α-TRIM32). Data are representative of three independent experiments. (D) Representative immunoblot of RAW 264.7 TRIM32 knockout (KO) cell whole-cell lysate. A clonal population of cells that went through the CRISPR knockout procedure unsuccessfully served as a negative control. Actin was used as the loading control. Data represent results of three independent experiments. (E) Representative images by confocal immunofluorescence microscopy of WT or TRIM32 KO RAW 264.7 macrophages infected with Salmonella strain Δ*sseK3*pSseK3-HA at 16 hpu: Salmonella (α-CSA-1, gray), effectors (α-HA, red), Golgi netwrk (α-Rab6, green), DNA (DAPI, blue). Bar, 5 μm.

Translocation of SseK1, SseK2, and SseK3 into HeLa cells was shown previously (24, 27). To analyze the involvement of the Salmonella SseK effectors on NF-κB signaling and host cell death during infection of macrophages, plasmids were created that expressed hemagglutinin (HA)-tagged SseK effectors under the control of their endogenous promoters. SPI-2 T3SS-dependent translocation of SseK1-HA, SseK2-HA, and SseK3-HA was detected in approximately 60% of infected RAW 264.7 macrophages at 16 h postuptake (hpu) (Fig. 1A and B; see also Fig. S1 in the supplemental material). Translocated SseK1-HA was diffusely cytosolic with no specific subcellular localization (Fig. 1A). In contrast, all cells positive for translocated SseK2-HA and SseK3-HA showed clear and well-defined colocalization of the effector with the host Golgi network (labeled with anti-Rab6 antibody) (Fig. 1A). This differential localization of SseK1 and SseK3 confirms previous studies that used ectopically expressed effectors after transfection (26, 27).

The E3-ubiquitin ligase TRIM32 is the only known host protein to interact with SseK3 (26). First, we tested if TRIM32 and the SseK effectors interacted during infection. HA-tagged SseK3, but not SseK1-HA or SseK2-HA, specifically bound endogenous TRIM32 in macrophage lysates prepared 16 h postuptake (Fig. 1C). TRIM32 localizes to cytosolic perinuclear speckles (28, 29) as well as to the Golgi network (26). To investigate if Golgi network localization of SseK3-HA during infection depends on TRIM32, we generated TRIM32 null macrophages through the CRISPR-Cas9 method (30, 31) (Fig. 1D; Fig. S2A). Translocation of SseK3-HA in TRIM32 knockout macrophages was indistinguishable from that in wild-type cells, occurring in approximately 70% of infected cells, with Golgi network localization of SseK3-HA detected in 100% of cells containing the effector (Fig. 1E). Therefore, TRIM32 is not required for the translocation or localization of SseK3.

### SseK1 and SseK3 inhibit the NF-κB pathway during Salmonella infection.

**FIG 2:**
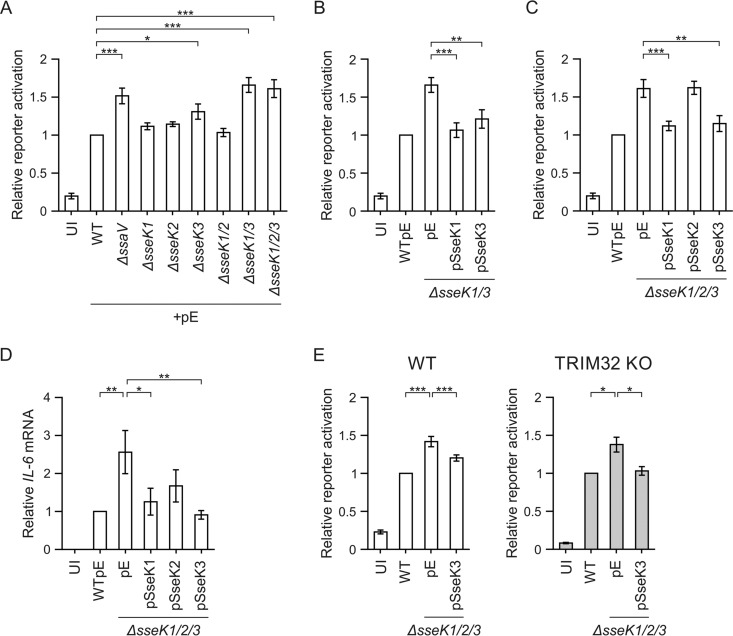
SseK1 and SseK3 inhibit the NF-κB pathway in an additive manner during macrophage infection. (A to C) RAW 264.7 macrophages stably transduced with an NF-κB-dependent luciferase gene and constitutively expressing Renilla luciferase as an internal control were infected for 16 h with the indicated Salmonella strains. Results are represented as the mean fold activation relative to that after wild-type infection from five independent experiments, ± SEM. *, *P* < 0.05; **, *P* < 0.01; ***, *P* < 0.001. All data were acquired at the same time. The UI, WTpE, Δ*sseK1/3*pE, and Δ*sseK1/2/3*pE data are reproduced in each panel for ease of interpretation. UI, uninfected; pE, empty control vector. (D) Quantitative RT-PCR analysis of *IL-6* mRNA levels in infected *Tlr4*^−/−^ iBMDMs at 16 hpu. *IL-6* mRNA expression was normalized to *Rps9* mRNA and is presented as the fold change relative to WT-infected cells. Results are presented as means from six independent experiments ± SEM. *, *P* < 0.05; **, *P* < 0.01. (E) WT or TRIM32 knockout (KO) RAW 264.7 NF-κB reporter macrophages were infected for 16 h with the indicated Salmonella strains. Results are presented as the mean fold activation relative to that after wild-type infection from three independent experiments, ± SEM. *, *P* < 0.05; ***, *P* < 0.001. UI, uninfected; pE, empty control vector.

To investigate the effects of the SseK proteins on the NF-κB pathway during Salmonella infection, we created a RAW 264.7 macrophage reporter cell line that stably expresses an NF-κB-dependent firefly luciferase gene and constitutively expresses Renilla luciferase as an internal control. These reporter cells were infected for 16 h with different Salmonella strains, and luciferase levels were measured. First, we confirmed that no replication defect of the mutant strains was apparent at this time point (32) (Fig. S3). Infection with wild-type Salmonella activated the reporter 5-fold compared to uninfected control cells (Fig. 2A), showing NF-κB pathway responsiveness of the cell line to Salmonella infection. The SPI-2 T3SS null mutant (Δ*ssaV* strain), previously described to elicit an increased host cell immune response in *Tlr4*^−/−^ macrophages (6), resulted in increased NF-κB reporter activity (Fig. 2A). To investigate if the loss of NF-κB pathway suppression by the Δ*ssaV* mutant strain was due to the lack of SseK effector translocation, we analyzed single, double, and triple *sseK* deletion mutant strains. There was significantly increased NF-κB pathway activity in cells infected with the Δ*sseK3*, Δ*sseK1* Δ*sseK3* (*ΔsseK1/3*), or Δ*sseK1* Δ*sseK2* Δ*sseK3* (Δ*sseK1/2/3*) strain compared to the wild-type strain response (Fig. 2A). Notably, the Δ*sseK1/3* double mutant strain had an additive effect compared to cells infected with either the Δ*sseK1* (*P* = 0.0001) or the Δ*sseK3* (*P* = 0.0144) strain. Decreased NF-κB suppression in macrophages infected with the Δ*sseK1/3* strain was complemented by expression of SseK1-HA or SseK3-HA from a low-copy-number plasmid (Fig. 2B). Additional deletion of *sseK2* (yielding the Δ*sseK1/2/3* triple mutant) did not increase NF-κB pathway activity above that with the Δ*sseK1/3* double mutant (Fig. 2A). Furthermore, in contrast to expression of SseK1-HA or SseK3-HA, expression of SseK2-HA from a low-copy-number plasmid did not suppress the increase in NF-κB signaling of the Δ*sseK1/2/3* triple mutant (Fig. 2C). Next, we analyzed activation of endogenous interleukin 6 (IL-6), an NF-κB-regulated cytokine, during Salmonella infection. To reduce the potent effect of lipopolysaccharide (LPS) on proinflammatory cytokine expression, which can mask the effect of SPI-2 T3SS-translocated proteins (6), we used *Tlr4*^−/−^ iBMDMs (Toll-like receptor 4 knockout immortalized bone marrow-derived macrophages). Expression of SseK1-HA or SseK3-HA in the Δ*sseK1/2/3* triple mutant resulted in significantly decreased *IL-6* mRNA levels compared to those for cells infected with the Δ*sseK1/2/3*pEmpty strain (Fig. 2D). Together, these results show that SseK1 and SseK3 function additively to inhibit the NF-κB signaling pathway during Salmonella infection.

### TRIM32 is not required for SseK3-mediated inhibition of NF-κB signaling.

Knockdown of TRIM32 decreases TNF-α-driven NF-κB signaling (26), suggesting that TRIM32 might function as an enhancer of NF-κB activation. To test directly if SseK3 interferes with NF-κB signaling in a TRIM32-dependent manner, we introduced the NF-κB-dependent luciferase reporter and Renilla luciferase genes into macrophages lacking TRIM32. Analysis of bacterial uptake (Fig. S2B) and replication by flow cytometry (Fig. S2C) or CFU assay (Fig. S2D) did not reveal any significant differences between wild-type Salmonella and the Δ*sseK1/2/3* triple mutant in RAW 264.7 macrophages, a CRISPR control clone, or the TRIM32 knockout cells. As was observed for RAW 264.7 macrophages, TRIM32 knockout cells infected with Δ*sseK1/2/3*pEmpty displayed increased NF-κB-dependent luciferase activity compared to cells infected with wild-type Salmonella or the Δ*sseK1/2/3* mutant expressing SseK3 from a plasmid (Fig. 2E). Therefore, TRIM32 is not required for SseK3 to inhibit NF-κB signaling during infection of macrophages.

### Specific inhibition of TNF-α-driven NF-κB signaling by SseK effectors.

**FIG 3:**
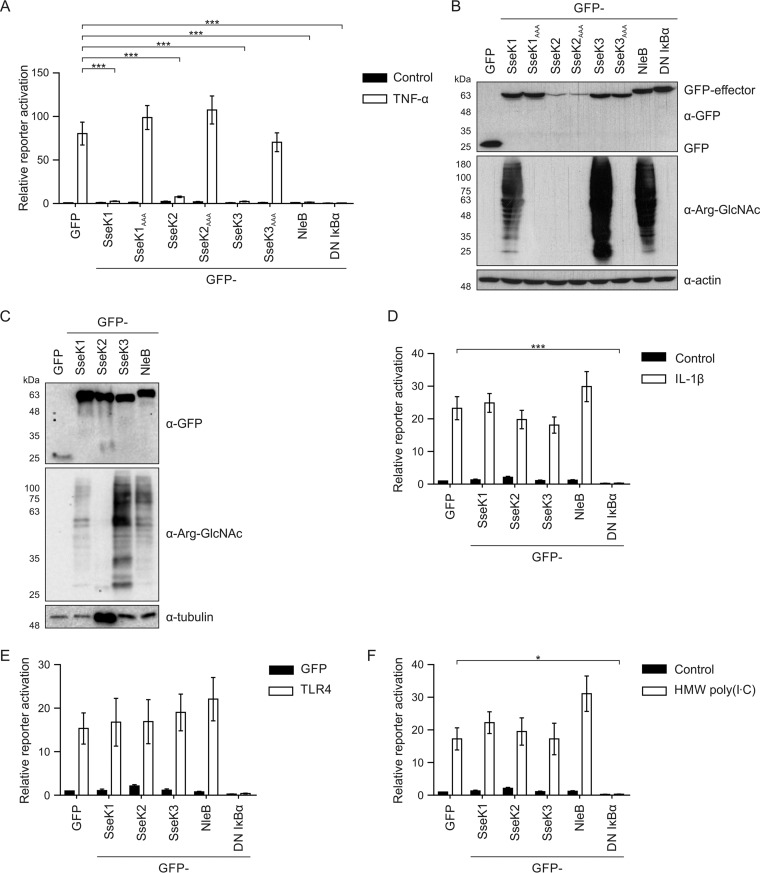
Salmonella SseK effectors selectively inhibit TNF-α-mediated NF-κB signaling. (A, D, E, and F) 293ET cells were cotransfected with an NF-κB-dependent luciferase reporter plasmid, pTK-Renilla luciferase plasmid, and the indicated ptCMV-GFP-effector plasmids. Alternatively, dominant negative IκBα was used as a control. The NF-κB pathway was activated overnight with 50 ng/ml TNF-α (A), 10 ng/ml IL-1β (D), 4 μg/ml HMW poly(I·C) (F), or overexpression of TLR4 for 24 h (E) prior to cell lysis and analysis of luciferase activity. Results shown in panels D and F were acquired at the same time and contain the same unstimulated control data. Results are presented as the fold activation relative to activation of unstimulated, GFP-expressing control cells. Data shown are means of 4 to 7 independent experiments ± SEM. *, *P* < 0.05; ***, *P* < 0.001. (B) Cell lysates from the experiment shown in panel A were analyzed by SDS-PAGE and immunoblotting to determine effector protein expression levels during NF-κB transfection reporter experiments. Effectors were anti-GFP (α-GFP), anti-arginine-GlcNAc (α-Arg-GlcNAc), and loading controls (α-actin and α-tubulin). (C) The amount of cell lysate was adjusted to normalize GFP-effector levels, and samples were analyzed as described for panel B.

It has been reported that SseK1 and SseK3 are sufficient to block TNF-α-driven NF-κB signaling when overexpressed in mammalian cells (21, 26). We confirmed these results: SseK1 and SseK3 inhibited the phosphorylation of IκBα after TNF-α stimulation (Fig. S4A) and inhibited TNF-α-induced NF-κB reporter activation (Fig. 3A). Mutation of the putative divalent cation and/or sugar-coordinating DXD motif to AAA caused loss of pathway inhibition, despite equivalent levels of expression of wild-type and mutant proteins (Fig. 3A and B). Despite only weak inhibition of IκBα phosphorylation after SseK2 expression (Fig. S4A), SseK2 inhibited the NF-κB reporter after TNF-α stimulation (Fig. 3A). Whether SseK2 is a bona fide inhibitor of NF-κB activation awaits additional work, but it is noteworthy that in our infection experiments we were unable to identify a phenotype for SseK2 (Fig. 2).

To better understand the specificity of NF-κB pathway inhibition, we measured reporter activation after effector plasmid transfection and pathway activation with a single stimulus. As a positive control, inhibition by dominant negative IκBα (S32A/S36A) was also analyzed. Proinflammatory cytokines of the IL-1 family activate a distinct signaling pathway to TNF-α that converges at the IκB kinase (IKK) complex upstream of NF-κB activation (15). As expected from previous work (33, 34), NleB was not an inhibitor of IL-1α- or IL-1β-induced NF-κB activation (Fig. 3D; Fig. S4B). Also, whereas dominant negative IκBα inhibited NF-κB activity following stimulation with IL-1α (Fig. S4B) or IL-1β (Fig. 3D), none of the SseK proteins had a significant effect. A major activator of NF-κB during infection is bacterial LPS. To mimic LPS stimulation in 293ET cells, the LPS receptor TLR4 was overexpressed to autoactivate the NF-κB signaling pathway. In contrast to TNF-α stimulation, SseK1, SseK2, and SseK3 did not inhibit NF-κB activation downstream of TLR4 (Fig. 3E). Intracellular RIG-I-like receptors (RLRs) activate NF-κB as well as the interferon-regulated factor (IRF) family of transcription factors (35). As Salmonella RNA can enter the cytosol, thereby activating RIG-I-dependent signaling (36), we tested if SseK effectors inhibit RLR-induced NF-κB signaling after stimulation with polyinosinic:polycytidylic acid [poly(I·C)], a double-stranded RNA (dsRNA) mimic. As expected, dominant negative IκBα inhibited NF-κB activation, but no inhibition occurred upon overexpression of green fluorescent protein (GFP)-tagged SseK1, SseK2, or SseK3 (Fig. 3F). Therefore, the SseK effectors appear to have a TNF-α-specific inhibitory effect on NF-κB signaling.

Inhibition of the NF-κB pathway by NleB depends on its arginine-GlcNAc transferase activity (21). We used SDS-PAGE and immunoblotting with an arginine-GlcNAc-specific antibody (37) to visualize arginine-GlcNAcylated proteins in cell lysates used for the reporter assay described above. Production of SseK1 and SseK3 as well as the positive control NleB resulted in a strong polydisperse smear of arginine-GlcNAcylated proteins (Fig. 3B). Loading less protein resolved individual bands representing arginine-GlcNAcylated proteins induced by SseK1 and SseK3 (Fig. 3C). Arginine-GlcNAcylation induced by SseK2 was only detected after normalization of effector protein levels and very long exposure (Fig. S4C). Expression of the putative catalytic mutants SseK1_AAA_ and SseK3_AAA_, which did not inhibit NF-κB activation (Fig. 3A), failed to induce arginine-GlcNAcylation (Fig. 3B). Therefore, SseK1- and SseK3-mediated inhibition of NF-κB activation is correlated with the arginine-GlcNAcylation of cellular proteins.

### SseK1 and SseK3 expression leads to different arginine-GlcNAcylation patterns.

**FIG 4:**
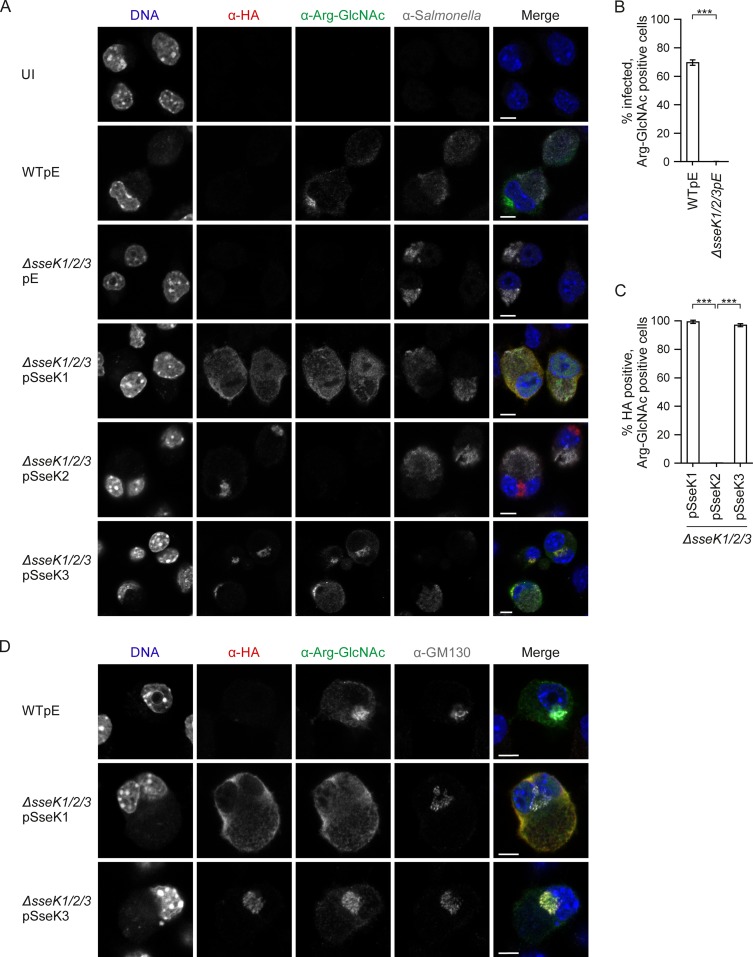
SseK-dependent differential localization of arginine-GlcNAcylated proteins in infected macrophages. (A) Representative confocal immunofluorescence microscopy images of arginine-GlcNAc patterns in RAW 264.7 macrophages infected with wild-type or the indicated mutant Salmonella strains expressing HA-tagged SseK effectors at 16 hpu. Images are for Salmonella (anti-CSA-1 [α-CSA-1], gray), effectors (α-HA, red), arginine-GlcNAc (α-Arg-GlcNAc, green), and DNA (DAPI, blue). Bar, 5 μm. (B) Percentage of infected cells with arginine-GlcNAcylated proteins, quantified from the results shown in panel A. (C) Percentage of infected cells with a translocated effector, showing a positive arginine-GlcNAc signal, quantified from the experiment shown in panel A. Data are mean results ± SEM of three independent experiments with at least 600 infected cells counted in total. ***, *P* < 0.001. (D) Representative images of arginine-GlcNAcylation and Golgi network (α-GM130, gray) colocalization in RAW 264.7 macrophages. Bar, 5 μm. UI, uninfected; pE, empty vector control.

Next, we studied arginine-GlcNAcylation induced by the SseK effectors under the more physiological conditions of infection. Salmonella-infected RAW 264.7 macrophages gave rise to an arginine-GlcNAcylation signal in the cytosol and at the Golgi network that was detected in approximately 70% of the wild-type-infected cells but completely absent in cells infected with the Δ*sseK1/2/3* mutant (Fig. 4A and B). The Δ*sseK1/2/3* mutant expressing SseK1 or SseK3 restored arginine-GlcNAcylation in the host cell cytoplasm (SseK1) or both in the cytoplasm and at the Golgi network (SseK3) (Fig. 4A and D). No arginine-GlcNAcylated proteins were detected in the Δ*sseK1/2/3*pSseK2 strain, despite clear translocation of the HA-tagged effector (Fig. 4A and C).

**FIG 5:**
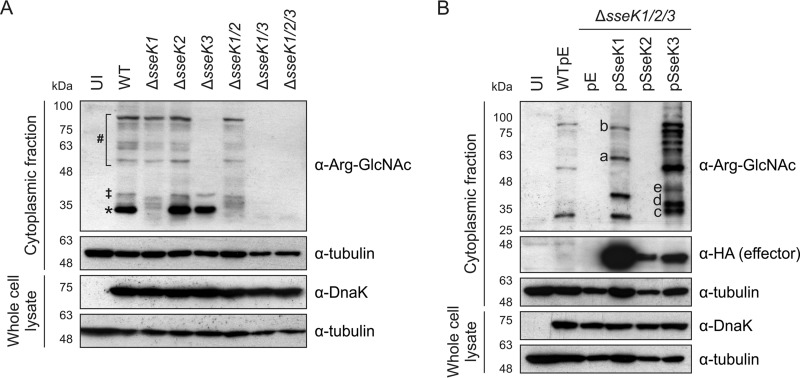
Expression of SseK1 and SseK3 results in different patterns of arginine-GlcNAcylated proteins during infection of macrophages. (A and B) RAW 264.7 macrophages were infected with the indicated Salmonella strains for 16 h. Postnuclear extracts were isolated, and arginine-GlcNAcylated proteins were analyzed by SDS-PAGE and immunoblotting. Where indicated, translocated HA-tagged effectors were also probed. Whole-cell lysates were analyzed to control for bacterial loads. Data include tests with antibodies against arginine-GlcNAc (α-Arg-GlcNAc), Salmonella (α-DnaK), effectors (α-HA), and the loading control (α-tubulin). Data are representative of results of three independent experiments. UI, uninfected; pE, empty vector control. * and ‡, SseK1-specific arginine-GlcNAcylated proteins; # and the brackets highlight a range of SseK3-specific arginine-GlcNAcylated proteins.

The differential subcellular localization of arginine-GlcNAcylated proteins upon expression of SseK1 or SseK3 suggested that these enzymes might target different substrates during infection. To test this directly, RAW 264.7 macrophages were infected for 16 h with different Salmonella strains, and proteins were analyzed by SDS-PAGE and immunoblotting. Several arginine-GlcNAcylated proteins were detected after infection with wild-type Salmonella (Fig. 5A and B). Deletion of *sseK2* produced no detectable effect on the arginine-GlcNAcylation pattern compared to that for wild-type-infected cells (Fig. 5A). In agreement with this, no arginine-GlcNAcylated proteins were detected after infection with the Δ*sseK1/3* mutant strain (Fig. 5A). Furthermore, and in line with the fluorescence microscopy (Fig. 4A), expression of SseK2 in the Δ*sseK1/2/3* mutant strain did not result in detectable arginine-GlcNAcylated proteins (Fig. 5B). In contrast, single deletion of *sseK1* or *sseK3* led to the loss of individual GlcNAcylated proteins on the immunoblot, suggesting effector-specific target proteins (Fig. 5A). Notably, a strong band corresponding to a protein of approximately 30 kDa (indicated by an asterisk) and a second protein of approximately 40 kDa (indicated by a double dagger) were absent in macrophages infected with the Δ*sseK1* but not the Δ*sseK2* or Δ*sseK3* strain (Fig. 5A). Overexpression of translocated SseK1 from a low-copy-number plasmid restored two bands with the same approximate molecular mass of the bands absent after infection with the Δ*sseK1* strain, indicating that they are physiological targets of SseK1 (Fig. 5A and B). Additional prominent bands at approximately 65 and 95 kDa (bands a and b, respectively) became apparent specifically when SseK1 was overexpressed from bacteria (Fig. 5B).

Following infection with the Δ*sseK3* strain, at least 6 discernible GlcNAcylated proteins from 55 to 100 kDa (indicated by a hash) were absent, compared to results for macrophages infected with wild-type Salmonella (Fig. 5A). GlcNAcylated proteins of corresponding molecular masses were restored when macrophages infected with the Δ*sseK1/2/3*pSseK3 strain were analyzed (Fig. 5B). In addition, 3 strong bands of approximately 32, 35, and 43 kDa (bands c, d, and e) were detected. It is noteworthy that the 9 strong bands detected upon plasmid-based expression of SseK3 are all of different molecular masses than those detected after infection with the Δ*sseK1/2/3*pSseK1 strain (Fig. 5B), strongly suggesting differential substrate specificities of these effectors.

### Analysis of potential targets.

**FIG 6:**
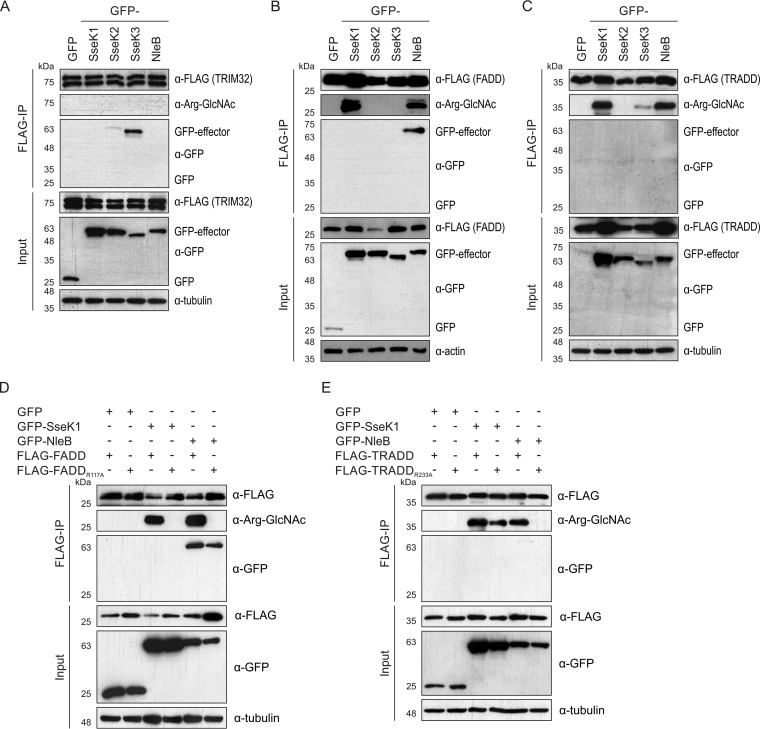
Arginine-GlcNAcylation profile of FADD and TRADD upon SseK expression. (A to E) Plasmids harboring GFP-tagged effectors and FLAG-tagged putative target proteins were cotransfected into 293ET cells. At 40 h after transfection, cells were lysed and proteins were immunoprecipitated with α-FLAG-conjugated beads. Samples were analyzed by SDS-PAGE and immunoblotting for effectors (α-GFP), TRIM32/FADD/TRADD (α-FLAG), arginine-GlcNAc (α-Arg-GlcNAc), and the loading control (α-tubulin or α-actin). Data are representative of three independent experiments.

Yang et al. (26) showed that despite stable binding of SseK3 to TRIM32, no GlcNAc modification of TRIM32 was detected when we used an antibody against O-GlcNAc posttranslational modifications. Similarly, despite binding of GFP-SseK3 to FLAG-TRIM32, we detected no arginine-GlcNAcylation of TRIM32 (Fig. 6A). Based on the high level of amino acid identity between NleB and the SseK effectors (24), we hypothesized that the SseK proteins might also arginine-GlcNAcylate death domain-containing proteins (21, 22). To test this, FLAG-tagged FADD or TRADD, two known NleB targets, was expressed together with SseK1, SseK2, or SseK3 in 293ET cells and enriched by anti-FLAG immunoprecipitation. As expected, NleB bound FADD stably and caused its arginine-GlcNAcylation (Fig. 6B). Even though stable binding between SseK1 and FADD was not detected, overexpression of the SseK1 resulted in arginine-GlcNAcylation of FADD (Fig. 6B). Modification of FADD was not detected when FADD was expressed in the presence of SseK2 or SseK3 (Fig. 6B). Similar to FADD, no stable effector binding to TRADD was detected, but the protein was GlcNAcylated when coexpressed with SseK1 or NleB, in line with previously reported findings (21). Very minor modification of TRADD was observed when SseK3 was coexpressed (Fig. 6C). These data demonstrated target specificity between SseK1 and SseK3, in line with our observation that different proteins are arginine-GlcNAcylated by these effectors during infection.

Next, we used arginine mutants of FADD and TRADD to test whether SseK1 and NleB induce GlcNAcylation of the same amino acid residue in these proteins. FADD_R117A_ was not GlcNAcylated upon expression of SseK1 or NleB, indicating that both effectors target the same residue in the death domain of FADD (Fig. 6D). In contrast, TRADD_R233A_ was still modified when coexpressed with SseK1 but not NleB, suggesting that SseK1-mediated GlcNAcylation of TRADD occurs either on a different amino acid or on multiple arginine residues (Fig. 6E).

**FIG 7:**
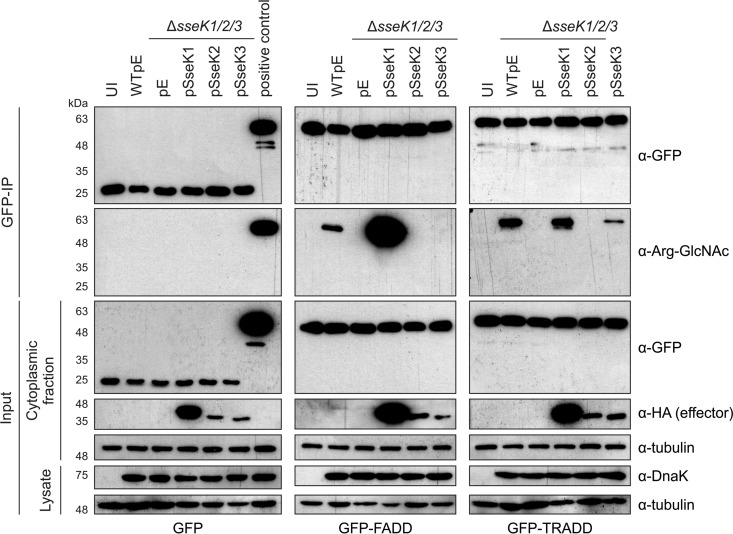
Arginine-GlcNAcylation profile of FADD and TRADD during Salmonella infection. Plasmids carrying genes for GFP (left panel), GFP-FADD (middle panel), and GFP-TRADD (right panel) were transfected into HeLa cells. At 24 h after transfection, cells were infected with the indicated Salmonella strains. After 16 h of infection, cells were lysed and proteins were immunoprecipitated with α-GFP-conjugated beads. Samples were analyzed by SDS-PAGE and immunoblotting for effectors (α-HA), FADD/TRADD (α-GFP), arginine-GlcNAc (α-Arg-GlcNAc), Salmonella (α-DnaK), and a loading control (α-tubulin). The positive control in the left panel is GFP-TRADD-transfected cells infected with WTpE Salmonella, as in the right panel. Data are representative of results from three independent experiments. UI, uninfected; pE, empty vector control.

Finally, we tested if FADD and TRADD were modified after bacterially mediated delivery of SseK proteins. Transiently expressed GFP-tagged FADD or TRADD, or GFP alone (as a control), was immunoprecipitated from noninfected HeLa cells or cells infected with WTpEmpty, Δ*sseK1/2/3*pEmpty, or Δ*sseK1/2/3* strains expressing SseK1, SseK2, or SseK3 (Fig. 7). Infection-induced arginine-GlcNAcylation of FADD and TRADD (but not of the GFP control) was dependent on the SseK proteins: SseK1 modified FADD (Fig. 7, middle panel) while both SseK1 and SseK3 induced modification of TRADD (Fig. 7, right panel). Therefore, FADD and TRADD are likely to represent genuine targets of SseK proteins during host cell infection.

### SseK1 and SseK3 inhibit cell death during Salmonella infection.

**FIG 8:**
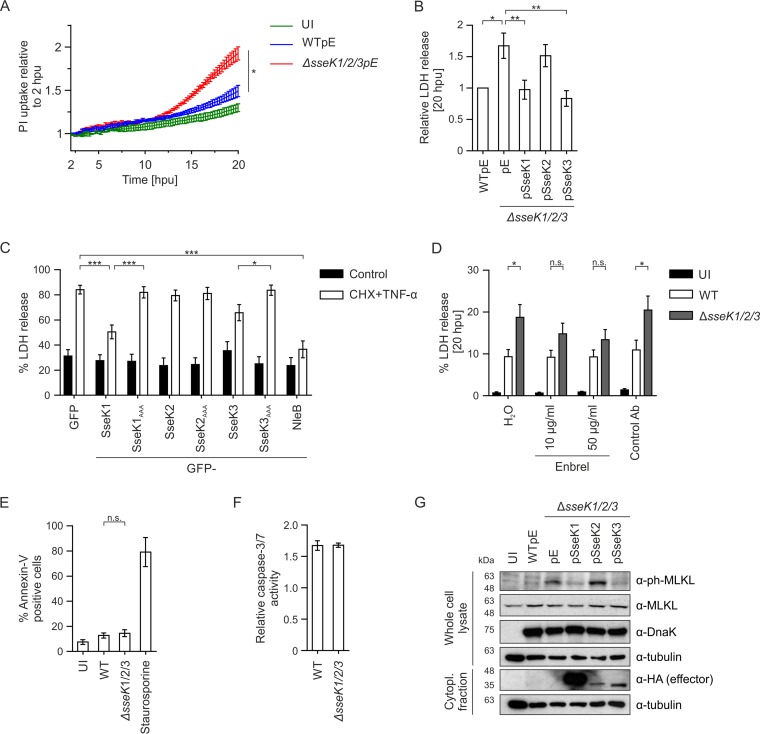
SseK1 and SseK3 inhibit TNF-α-induced cell death in macrophages. (A) RAW 264.7 macrophages were infected with wild-type or mutant Salmonella strains, and cell death was assayed based on PI uptake over time. Results from three independent experiments were calculated relative to maximum PI uptake and are presented as the mean fold increase relative to results at 2 hpu, ± SEM. *, *P* < 0.05. (B) Lactate dehydrogenase release from RAW 264.7 macrophages infected for 20 h with the indicated Salmonella strains. Salmonella-induced cell death was calculated and normalized to that for WT-infected cells. Data presented are the means of eight independent experiments, ± SEM. *, *P* < 0.05; **, *P* < 0.01. (C) LDH release from transfected HeLa cells expressing GFP-tagged SseK effectors. Cell death was stimulated with 10 μg/ml cycloheximide (CHX) and 50 ng/ml TNF-α for 20 h. Data presented are the mean results of nine independent experiments, ± SEM. *, *P* < 0.05; ***, *P* < 0.001. (D) LDH release from RAW 264.7 macrophages infected for 20 h with the indicated strains and treated with water, the TNF-α inhibitor etanercept (Enbrel), or an antibody (Ab) control used at 50 μg/ml. Data are normalized to results with uninfected, water-treated control cells and represent the means of six independent experiments, ± SEM. *, *P* < 0.05; n.s., not significant. (E) RAW 264.7 macrophages were infected for 20 h with the indicated GFP-expressing strains, and Annexin-V labeling of infected cells was analyzed by flow cytometry. Staurosporine (1 μM) was used as a positive control to induce apoptosis. Data are the mean results of three independent experiments, ± SEM. (F) Caspase-3/7 activity was assayed using an SR-DEVD-FMK FLICA probe at 20 hpu in macrophages infected with the indicated GFP-expressing strains. The data, which represent the mean results and SEM of three independent experiments, were analyzed by flow cytometry and are presented as the fold change in activity of infected versus uninfected cells from the same sample. (G) RAW 264.7 macrophages were infected with the indicated Salmonella strains for 20 h. MLKL phosphorylation and effector translocation were analyzed in whole-cell lysates and postnuclear supernatants by SDS-PAGE and immunoblotting. Phospho-MLKL (α-ph-MLKL), MLKL (α-MLKL), effectors (α-HA), Salmonella (α-DnaK), loading control (α-tubulin) were included. Data are representative of results from three independent experiments. UI, uninfected; Cytopl., cytoplasmic.

GlcNAcylation of FADD and TRADD by NleB results in inhibition of cell death as well as inhibition of NF-κB signaling (21, 22). We therefore tested if SseK proteins also inhibited cell death. First, we analyzed the uptake of propidium iodide (PI), a membrane-impermeable dye, by infected macrophages over time. Macrophages infected with wild-type Salmonella showed increased levels of PI uptake compared to uninfected cells, and this was further increased when cells were infected with the Δ*sseK1/2/3* mutant strain (Fig. 8A; Fig. S5A). Next, we analyzed cell death by release of lactate dehydrogenase (LDH) at 20 hpu, when significant differences in PI uptake between the Δ*sseK1/2/3* mutant and wild-type Salmonella were observed. LDH release was significantly increased in macrophages infected with the Δ*sseK1/2/3* mutant compared to the level in wild-type infected cells. Expression of SseK1 or SseK3 (but not SseK2) in the Δ*sseK1/2/3* mutant was sufficient to reduce LDH release (Fig. 8B; Fig. S5B), revealing that during infection both SseK1 and SseK3 inhibit Salmonella-induced cell death.

Similar to NleB (21), SseK1 and SseK3 inhibited TNF-α plus cycloheximide-induced apoptosis in HeLa cells (Fig. 8C; Fig. S5C). Therefore, we tested if SseK effectors inhibit TNF-α-induced cell death during infection of macrophages. First, we confirmed that pharmacological inhibition of extracellular TNF-α (using etanercept [Enbrel], which binds to TNF-α [38–40]) reduced TNF-α-mediated activation of the NF-κB reporter in a dose-dependent manner (Fig. S5D). The addition of Enbrel, which did not affect the uptake (Fig. S5E) or replication (Fig. S5F) of Salmonella, abrogated the increased LDH release that is normally detected in macrophages infected with Δ*sseK1/2/3* mutant bacteria (Fig. 8D). Therefore, during infection, exogenous TNF-α induces cell death in the absence of SseK-mediated inhibition.

Activation of the TNFR can induce apoptosis or necroptosis, depending on the amount of caspase activity (41, 42). To test if macrophages infected with the Δ*sseK1/2/3* mutant Salmonella strain undergo enhanced apoptosis, we determined the proportion of cells positive for Annexin-V binding (it binds phosphatidylserine on the extracellular surface of apoptotic cells) by flow cytometry. Approximately 15% of infected cells were Annexin-V positive, whether the bacteria expressed SseK proteins or not (Fig. 8E). Death receptor signaling activates caspase-8, which cleaves and activates caspase-3 during apoptosis. Therefore, as an alternative readout for the induction of apoptosis, we measured the amount of caspase-3/-7 activity in infected macrophages. No differences in caspase-3/-7 activity were detected after 20 h of infection with either wild-type or Δ*sseK1/2/3* mutant Salmonella cells (Fig. 8F; Fig. S5G). In contrast to apoptosis, necroptotic cell death depends on the phosphorylation and activation of MLKL (18–20). In comparison to wild-type Salmonella-infected macrophages, those infected with the Δ*sseK1/2/3* mutant exhibited strikingly enhanced levels of phospho-MLKL (Fig. 8G). The enhanced levels of phospho-MLKL were reduced in Δ*sseK1/2/3* strains expressing SseK1 or SseK3, but not those expressing SseK2. Together, our data indicate that during macrophage infection SseK1 and SseK3 function to inhibit TNF-α-induced necroptotic cell death.

## DISCUSSION

Two comprehensive studies showed that the arginine-GlcNAcylation of death domain proteins, including TRADD and FADD, by the E. coli effector protein NleB results in inhibition of NF-κB signaling and cell death (21, 22). The Salmonella genome encodes three proteins with high amino acid identity to NleB (24). By using mutant strains and transient transfection to analyze the function of SseK proteins, we investigated their relative contributions to immune regulation. We found that during macrophage infection, SseK1 and SseK3 inhibited NF-κB signaling in an additive manner and also suppressed host cell death. Ectopic overexpression of SseK2 resulted in inhibition of TNF-α-induced NF-κB reporter activation, which was dependent on its DXD motif, indicating that it mediates this effect enzymatically. However, whether this activity is nonspecific or of physiological relevance is unclear, as we were unable to detect altered NF-κB induction or arginine-GlcNAcylation by SseK2 during infection of macrophages with Salmonella, despite clear evidence of effector translocation. Therefore, while it remains conceivable that GlcNAcylated proteins induced by SseK2 during infection are below the level of detection, it is also possible that this effector does not act as an arginine-GlcNAc transferase within macrophages. SseK2 might have evolved to preferentially catalyze the GlcNAcylation of a different amino acid residue(s) or to transfer a different saccharide moiety that is not detected by the antibody used here. Whatever its biochemical activity, inhibition of NF-κB activation during mouse macrophage infection seems unlikely to be the primary function of SseK2.

In contrast to SseK2, both SseK1 and SseK3 induced specific and distinct arginine-GlcNAcylation of several proteins during infection of macrophages. Given the high degree of similarity between SseK proteins and NleB (in terms of amino acid sequence, conservation of the essential DXD motif, and functional inhibition of TNF-α-induced NF-κB signaling), we tested if SseK effectors also induce the arginine-GlcNAcylation of TRADD and FADD. We confirmed a previous finding that SseK1 induces GlcNAcylation of overexpressed TRADD (21), and we also detected SseK1-dependent arginine-GlcNAcylation of FADD, despite the lack of stable binding between SseK1 and these host proteins. Like NleB, SseK1 induced GlcNAcylation of R117 on FADD, indicating that it has the same activity. In contrast to NleB, SseK1 still promoted arginine-GlcNAcylation of a TRADD variant in which the arginine residue modified by NleB (R233) was mutated. Evidently, SseK1 modifies either different or multiple arginine residues in TRADD. SseK1-mediated modification of overexpressed TRADD and FADD was detected in HeLa cells after translocation of the effector from bacteria. After macrophage infection, several proteins of a similar molecular weight as TRADD were GlcNAcylated in an SseK1-dependent manner (Fig. 5A), but which (if any) of these represent TRADD remains to be established.

In contrast to SseK1 and NleB, ectopic expression of SseK3 did not induce detectable arginine-GlcNAcylation of FADD and caused much weaker TRADD modification than either SseK1 or NleB. In support of SseK3-mediated modification of TRADD, bacterially delivered SseK3 modified overexpressed TRADD in HeLa cells. The only known host protein that binds to SseK3 (upon ectopic expression) is the E3-ubiquitin ligase TRIM32 (26), and we established that this interaction also occurs in infected macrophages. Nearly half of all TRIM proteins are innate immune enhancers, and TRIM32 can bind/modify several proteins involved in NF-κB signaling, including IRAK1, PIASy, and STING (43–45), making TRIM32 a plausible candidate for SseK3-mediated GlcNAcylation. However, no modification of TRIM32 by SseK3 was detected by Yang et al. (26) when they used an anti-*O*-GlcNAc antibody or when we used an anti-arginine-GlcNAc-specific antibody (this study). As TRIM32 can self-associate into tetramers (46), a second hypothesis was that it might function as a structural scaffold for SseK3 function rather than being a direct effector target. However, SseK3 still inhibited NF-κB activation during infection of macrophages lacking TRIM32. Therefore, the physiological significance of the SseK3-TRIM32 interaction remains enigmatic.

A double mutant strain lacking SseK1 and SseK3 produced significantly more NF-κB reporter activation than either single mutant, suggesting that these effectors are not functionally redundant. Furthermore, the bacterial mutants and complemented strains produced patterns of GlcNAcylation on Western blot assays that enabled at least two proteins (approximately 30 and 40 kDa) to be assigned to SseK1 and at least six proteins (of 55 to 100 kDa) to be assigned to SseK3. The identities of these proteins are currently unknown but they clearly indicate distinct targets for SseK1 and SseK3. Since the effectors did not inhibit NF-κB signaling induced by TLR4, IL-1α/β, or poly(I·C), we concluded that at least some of the putative targets are likely to be components of the TNF-α-specific arm of this pathway.

During infection, rapid NF-κB activation occurs upon recognition of LPS by TLR4 (47). In the mouse model of infection, the influence of TNF-α in controlling bacterial growth only becomes apparent several days after inoculation (48). Since SPI-2 T3SS effectors are expressed and translocated by intracellular bacteria, following vacuole acidification and assembly of the secretion apparatus (49–51), it is not surprising that the SseK family has evolved to inhibit TNF-α- but not TLR4-induced signaling. This agonist-specific inhibition appears to distinguish SseK1 and SseK3 from other NF-κB-inhibiting effectors of Salmonella. These include GtgA, GogB, and SpvD, which target proteins common to numerous agonists (3, 6, 7).

Finally, we have shown that SseK1 and SseK3 inhibit necroptotic cell death during macrophage infection. Whether they inhibit apoptotic cell death during infection in cells that undergo higher levels of apoptosis awaits further investigation. The inhibition of death domain-containing proteins such as FADD and TRADD provides an explanation for how SseK1 and possibly SseK3 inhibit TNF-α-induced cell death in addition to NF-κB activation. Inhibition of both proinflammatory signaling and host cell death by SseK1 and SseK3 could provide Salmonella with robustness and flexibility in counteracting host immune responses.

## MATERIALS AND METHODS

### Bacterial strains and growth conditions.

The Salmonella Typhimurium strains (wild-type NCTC 12023 and its mutant derivatives) used in this study are listed in Table S1 in the supplemental material. For flow cytometry, the indicated bacterial strains expressed GFP from pFPV25.1. Bacteria were grown in LB broth at 37°C in an orbital shaker. When appropriate, cultures were supplemented with 50 μg/ml carbenicillin or 50 μg/ml kanamycin.

### Plasmid construction.

All plasmids and primers used in this study are detailed in Tables S2 and S3. *S.* Typhimurium mutant strains were complemented with pWSK29 derivative plasmids (52). SseK1, SseK2, and SseK3 and their upstream promoter regions (200 bp, 200 bp, and 1000 bp, respectively) were amplified from *S.* Typhimurium 12023 genomic DNA by PCR and ligated into pWSK29-2HA (53) by using EcoRI and BamHI restriction sites.

For effector expression in mammalian cells, the open reading frames of SseK1, SseK2, and SseK3 were amplified from *S.* Typhimurium 12023 genomic DNA by PCR. Following sequence alignment with E. coli NleB, putative catalytic DAD motifs of the SseK proteins were identified. The SseK_AAA_ constructs were created by using overlap-PCR with specific mutagenesis primers (54). NleB was amplified from genomic DNA of the E. coli O157:H7 Sakai strain (using primers adapted from those described in reference 22). Full-length FADD, TRADD, and TRIM32 were amplified from murine cDNA. FADD_R117A_ (primers described in reference 22) and TRADD_R233A_ were created by overlap-PCR. Human dominant negative (DN) IκBα (S32A/S36A) was described previously (55) (the template was a gift from F. Randow). The genes were cloned into the mammalian expression plasmid m4pGFP or m6pPAC-FLAG (both gifts from F. Randow) and resulted in proteins with an N-terminal GFP or FLAG tag, respectively. Renilla luciferase was amplified from pRLTK and ligated into m6pPAC without a tag. The ptCMV-GFP plasmid (this study) is a derivative from the pEGFP-N1 plasmid (Clontech). All plasmids were checked by sequencing.

### Cell lines and culture.

RAW 264.7 macrophages and HeLa cells used in this study were obtained from the European Collection of Animal and Cell Cultures (Salisbury, UK). 293ET cells were a gift from Felix Randow. Cells were cultured in Dulbecco's modified Eagle's medium (DMEM; Sigma) supplemented with 10% fetal calf serum (FCS; Sigma) at 37°C in 5% CO_2_. *Tlr4*^−/−^ immortalized BMDMs were created by infection of L929-differentiated bone marrow-derived macrophages (kind gift from Maria Belvisi and Mark Birrell, Imperial College London) with the v-*myc*/v-*raf*-expressing J2 retrovirus (56). Cells were then maintained in DMEM, 10% FCS, 20% L929 macrophage colony-stimulating factor and 1 mM sodium pyruvate at 37°C, 5% CO_2_. NF-κB reporter macrophages were generated by retroviral transduction of RAW 264.7 macrophages. Virus containing the m3psinrevκB-*luc* plasmid or the m6pPAC-RLuc plasmid was used for transduction as described previously (57). Cells were simultaneously transduced with both viruses and subsequently selected with puromycin (1.5 μg/ml).

### Etanercept inhibition.

To inhibit TNF-α stimulation of cells, RAW 274.7 macrophages were preexposed to 10 or 50 μg/ml etanercept (Enbrel; Pfizer) for 30 min and subsequently infected with the indicated Salmonella strains. Exposure of cells to the drug was maintained throughout the full duration of the experiments.

### Flow cytometry.

RAW 264.7 macrophages were infected in 12-well plates with GFP-expressing Salmonella strains. After 2 h, 16 h, or 20 h of infection, cells were washed with phosphate-buffered saline (PBS) and detached using Accutase (Sigma) for 15 min at room temperature. Cells were subsequently diluted in Opti-MEM (Invitrogen), and the GFP fluorescence intensity per cell was analyzed using a FACSCalibur flow cytometer (BD Biosciences). The fold change in bacterial replication was calculated by comparing the GFP geometric means (Flowing Software version 2.5.1) of the 2-h and 16-h time points. To analyze caspase-3/-7 activity, RAW 264.7 macrophages were infected for 20 h, and caspase activity was assayed using the SR-FLICA Caspase 3 and 7 assay kit (ImmunoChemistry Technologies) according to the manufacturer's recommendations. Cells treated for 20 h with 50 μg/ml cycloheximide (Sigma) and 50 ng/ml TNF-α (Sigma) were used as a positive control. Data were acquired using a Fortessa flow cytometer (BD Biosciences) and analyzed with FlowJo (version 8.8.6). Apoptotic RAW 264.7 macrophages were analyzed at 20 h post-bacterial uptake by using the Annexin-V apoptosis detection kit APC (eBioscience) according to the manufacturer's recommendations. Staurosporine (Sigma) at 1 μM for 20 h was used as a positive control. Data were acquired on a FACSCalibur apparatus (BD Biosciences) and analyzed with FlowJo software (version 8.8.6). For all flow cytometry experiments, data are the mean results of three independent experiments with a minimum of 10,000 (infected) cells analyzed in duplicate per sample.

### *In vitro* infection of cells.

RAW 264.7 macrophages were seeded at a density of 3 × 10^4^ cells per well (96-well plate), 1 × 10^5^ cells per well (24-well plate), 3 × 10^5^ cells per well (12-well plate), or 6 × 10^5^ cells per well (6-well plate) 24 h prior to use. Stationary-phase Salmonella cultures were opsonized in DMEM with 10% mouse serum for 20 min prior to macrophage infection (multiplicity of infection [MOI] of 10:1) by centrifugation for 5 min at 110 × *g* and incubation for 25 min at 37°C. For SPI-1-mediated invasion of HeLa cells, stationary-phase Salmonella strain cultures were subcultured (1:33) for 3.5 h at 37°C, and HeLa cells (3 ×10^5^ cells/well, 6-well plate) were exposed to 25 μl of subculture for 15 min. Cells were then washed in PBS, and extracellular bacteria were killed with medium containing 100 μg/ml gentamicin for 1 h. Subsequently, the gentamicin concentration was reduced to 20 μg/ml for the remainder of the infection. For analysis of arginine-GlcNAcylated proteins and anti-HA immunoprecipitation experiments, 1 × 10^7^ RAW 264.7 macrophages were seeded in 15-cm-diameter dishes 24 h before use. The macrophages were infected with stationary-phase Salmonella cultures at an approximate MOI of 30:1 for 30 min at 37°C. Subsequently, the cells were washed and treated with gentamicin as described above.

### Immunofluorescence microscopy.

After 16 h of RAW 264.7 macrophage infection, cells were fixed with 4% paraformaldehyde (PFA) for 20 min at room temperature. Cells were subsequently washed with PBS, and the PFA was quenched with 100 mM NH_4_Cl for a minimum of 1 h at 4°C. Coverslips were labeled with the indicated primary antibodies for 2 h at room temperature, washed with PBS, and incubated with the secondary antibodies and 4′,6-diamidino-2-phenylindole (DAPI) for 1 h at room temperature. All antibodies were diluted in PBS, 0.1% (vol/vol) Triton X-100 (or saponin, for the experiment shown in Fig. 1A) and 10% horse serum. Goat anti-Salmonella antibody (CSA-1; Kirkegaard and Perry Laboratories), rat anti-HA (3F10; Roche), mouse anti-GM130 (BD Biosciences), rabbit anti-Rab6 (C-19; Santa Cruz Biotechnology), and rabbit anti-arginine-GlcNAc (EPR18251; Abcam) were used. The Alexa Fluor 488-, 555-, and 633-conjugated donkey anti-goat, anti-rat, anti-mouse, and anti-rabbit secondary antibodies were purchased from Life Technologies, UK. Coverslips were mounted using Aqua-Poly/Mount (Polysciences, Inc.) and imaged using an LSM 710 inverted confocal microscope (Zeiss GmbH). For quantitative analysis, at least three independent experiments were performed in technical duplicates, and a minimum of 100 (infected) cells per coverslip were scored on an epifluorescence microscope (BX50; Olympus).

### NF-κB luciferase reporter assay.

NF-κB reporter macrophages were seeded in 24-well plates and infected as described above. After 16 h, cells were lysed in 100 μl passive lysis buffer (Promega). Alternatively, 5 × 10^4^ 293ET cells were seeded in 24-well plates 24 h prior to use. Transfection with Lipofectamine 2000 (Invitrogen) was performed according to the manufacturer's recommendations with a mixture of 20 ng pRLTK, 50 ng p4kB:Luc, and 250 ng ptCMV-GFP-effector (or pEGFP-N1 control) plasmids. At 24 h after transfection, cells were stimulated overnight (approximately 17 h) with 50 ng/ml TNF-α (Sigma), 10 ng/ml IL-1α (Sigma), 10 ng/ml IL-1β (Sigma), or 4 μg/ml high-molecular-weight (HMW) poly(I·C) (Invitrogen) and subsequently harvested in 100 μl passive lysis buffer (Promega). To autoactivate the NF-κB pathway, cells were transfected with 300 ng pEAKMMP-AU1-TLR4 (or m6pPAC-FLAG-GFP control) vector in addition to the plasmids described above for 24 h prior to harvesting cell lysates. Luciferase activity was measured using a dual luciferase reporter assay system (Promega) and a Tecan Infinite200 PRO plate reader. NF-κB-regulated luciferase activity was normalized to Renilla luciferase intensity and the results are presented relative to either wild-type-infected samples or unstimulated, GFP-expressing control samples.

### Cell fractionation.

RAW 264.7 macrophages were infected for 16 h, harvested, and lysed for 30 min on ice in lysis buffer (150 mM NaCl, 0.3% [vol/vol] Triton X-100, 20 mM Tris-Cl [pH 7.4], 5% glycerol, 5 mM EDTA, 1 mM phenylmethylsulfonyl fluoride, 1 mM benzamide, 2 μg/ml aprotinin, 5 μg/ml leupeptin, 1 mM dithiothreitol). A fraction of the whole-cell lysate was collected for analysis by SDS-PAGE and immunoblotting. The remainder of the sample was centrifuged for 30 min at 16,000 × *g* at 4°C. The postnuclear supernatant, containing cytoplasmic proteins, was separated from the pellet and analyzed by SDS-PAGE and immunoblotting.

### Immunoprecipitation experiments.

293ET cells were seeded in 6-well plates 24 h prior to use. One microgram of m6pPAC-FLAG-FADD, m6pPAC-FLAG-TRADD, or m6pPAC-FLAG-TRIM32 plasmid DNA together with 1 μg m6pPAC-FLAG-GFP, m4pGFP-SseK1, m4pGFP-SseK3, or m4pGFP-NleB or 200 ng ptCMV-GFP-SseK2 was transfected using Lipofectamine 2000 (Invitrogen) according to the manufacturer's instructions. After 40 h, cells were harvested and lysed in lysis buffer, and the postnuclear supernatant was isolated (see above). Proteins were immunoprecipitated using anti-FLAG M2 affinity gel (Sigma) for 2 h at 4°C. Samples were subsequently washed and analyzed by SDS-PAGE and immunoblotting. For infection anti-HA immunoprecipitation experiments, RAW 264.7 cells were seeded and infected for 16 h and proteins were isolated as described above. Anti-HA agarose (Pierce) was used to immunoprecipitate proteins, and beads were subsequently washed and analyzed by SDS-PAGE and immunoblotting. For immunoprecipitations following transfection and infection, HeLa cells were transfected for 24 h with 1 μg of m6pPAC-FLAG-GFP, m4pGFP-FADD, or m4pGFP-TRADD plasmid using Lipofectamine 2000 (Invitrogen). Cells were then infected for 16 h with the indicated Salmonella strains. Proteins were isolated as described above, immunoprecipitated using anti-GFP-Trap_A beads (ChromoTek), and analyzed by SDS-PAGE and immunoblotting.

### LDH release assay.

RAW 264.7 macrophages were infected in a 24-well plate as described above. After 30 min of infection, cells were then washed in PBS and extracellular bacteria were killed in phenol red-free medium containing 100 μg/ml gentamicin for 1 h. The medium was diluted to a final concentration of 20 μg/ml gentamicin for the remainder of the infection (20 h). To analyze TNF-α-induced cell death, HeLa cells were transfected with 250 ng ptCMV-GFP-effector (or pEGFP-N1 control) plasmids for 24 h by using Lipofectamine 2000 (Invitrogen). Cell death was subsequently stimulated with 10 μg/ml cycloheximide (Sigma) and 50 ng/ml TNF-α (Sigma) in phenol red-free medium. After 20 h of drug treatment or infection, the supernatant was collected and extracellular lactate dehydrogenase levels were measured with the CytoTox 96 nonradioactive cytotoxicity assay kit (Promega) according to the manufacturer's recommendations. Absorbance at 490 nm was measured on a Tecan Infinite200 Pro plate reader. Salmonella-induced cell death was calculated relative to that caused by the total cell death control (max).

### Quantitative RT-PCR.

Total RNA was isolated from 1 × 10^6^ *Tlr4*^−/−^ iBMDMs 16 hpu with the indicated bacterial strains (Qiagen RNAeasy minikit). Total RNA (400 ng) was used to synthesize cDNA according to the manufacturer's recommendations (QuantiTect RT kit, Qiagen). Technical duplicate quantitative reverse transcriptase PCRs (RT-PCRs; Sybr green PCR master mix; Applied Biosystems) were prepared containing 0.2 μM gene-specific primers and 0.5 μl cDNA. Emission data and a significant cycle threshold (*C_T_*) value from the Sybr green reporter dye were acquired on a Rotor-Gene 3000 (Corbett Research). Relative mRNA was calculated from a titration curve of cDNA. Data represent the relative amounts of *IL-6* mRNA, normalized to the amount of the *Rps9* housekeeping gene. The following primers were used: IL-6 (forward, 5′-AGACAAAGCCAGAGTCCTTCAG-3′, and reverse, 5′-GGTCTTGGTCCTTAGCCACTC-3′); Rps9 (forward, 5′-CTGGACGAGGGCAAGATGAAGC-3′, and reverse, 5′-TGACGTTGGCGGATGAGCACA-3′).

### Propidium iodide uptake.

RAW 264.7 macrophages were infected in a 96-well plate as described above. After 30 min of infection, cells were washed in PBS and incubated with medium (Opti-MEM, 10% FCS) containing 30 μg/ml gentamicin. Propidium iodide (used at 1:1,000; Sigma) uptake was then measured on a Tecan Infinite 200 Pro plate reader (excitation at 530 nm, emission at 617 nm) at 37°C in 5% CO_2_ for up to 20 hpu. PI uptake was calculated relative to that of a maximum control and normalized to findings at 2 hpu to account for experimental variation. Values shown are the means of three independent experiments (± standard errors of the means [SEM]).

### SDS-PAGE and immunoblotting.

Samples were separated on 10% SDS-PAGE gels and transferred onto polyvinylidene difluoride Immobilon-P membranes (Millipore). Membranes were blocked and incubated with primary antibodies overnight at 4°C, washed, and incubated for 2 h with horseradish peroxidase (HRP)-coupled secondary antibodies at room temperature. All antibodies were diluted in 5% nonfat milk in TBST (100 mM Tris-Cl [pH 7.4], 150 mM NaCl, 0.1% [vol/vol] Tween 20). Mouse anti-DnaK (8E2/2; Enzo), mouse anti-HA.11 (16B12; Covance), mouse anti-IκBα (L35A5; Cell Signaling), rabbit anti-IκBα phospho-S32 (14D4; Cell Signaling), rabbit anti-FLAG (Sigma), rabbit anti-GFP (Invitrogen), rabbit anti-arginine-GlcNAc (EPR18251; Abcam), rabbit anti-MLKL (Sigma), rabbit anti-MLKL phospho-S345 [EPR9515(2); Abcam], rabbit anti-TRIM32 (Abcam), rabbit anti-tubulin (EPR16774; Abcam), and rabbit anti-actin (Sigma) were used. The HRP-conjugated goat anti-mouse and anti-rabbit secondary antibodies were purchased from Dako. The immunoblots shown are representative of three independent experiments.

### TRIM32 CRISPR-Cas9.

RAW 264.7 macrophages were nucleofected (Lonza kit V) as per the manufacturer's recommendations with 2 μg of plasmid pX330:T2A-GFP (kind gift from F. Randow) carrying the guide RNA targeting *Trim32* (GACATTCTAGCACTTCCCGGAGG). Two days after transfection, cells were seeded in 96-well plates at a density of 0.3 cells/well. Clonal populations were screened for expression of TRIM32 by Western blotting. Sanger sequencing confirmed genome editing of *Trim32* in a clone lacking TRIM32 expression.

### Statistical analysis.

All presented values are expressed as means ± SEM of results from least three independent experiments. Statistical analysis was performed using Student's *t* test (two-tailed, unpaired) to compare two experimental groups. When comparing multiple groups, statistical significance levels were calculated using one-way analysis of variance (ANOVA) and a *post hoc* Dunnett test (*, *P* < 0.05; **, *P* < 0.01; ***, *P* < 0.001).

## Supplementary Material

Supplemental material
