# Supplementary material for: SseK1 and SseK3 Type III Secretion System Effectors Inhibit NF-κB Signaling and Necroptotic Cell Death in Salmonella-Infected Macrophages
Source: Infect Immun. 2017 Feb 23;85(3):e00010-17. doi: 10.1128/IAI.00010-17 (PMC5328493; doi:10.1128/IAI.00010-17)
Supplement: Supplemental material [file supp_85_3_e00010-17__index.html]

Supplemental material 

# SseK1 and SseK3 Type III Secretion System Effectors Inhibit NF-κB Signaling and Necroptotic Cell Death in Salmonella-Infected Macrophages

## Supplemental material

- Supplemental file 1 -

  Fig. S1. SPI-2 T3SS-dependent translocation of SseK effectors into macrophages. Fig. S2. Analysis of the TRIM32 knockout RAW 264.7 macrophages. Fig. S3. SseK deletion strains do not have a replication defect in macrophages. Fig. S4. *Salmonella* SseK effectors inhibit TNF-α-mediated IκBα phosphorylation but do not inhibit IL-1α-mediated NF-κB signaling. Fig. S5. SseK effectors inhibit TNF-α-driven cell death. Table S1. *S.* Typhimurium 12023 strains. Table S2. Plasmids. Table S3. Cloning primers.

  PDF, 2.8M
