# Supplementary material for: SseK1 and SseK3 Type III Secretion System Effectors Inhibit NF-κB Signaling and Necroptotic Cell Death in Salmonella-Infected Macrophages
Source: Infect Immun. 2017 Feb 23;85(3):e00010-17. doi: 10.1128/IAI.00010-17 (PMC5328493; doi:10.1128/IAI.00010-17)
Supplement: Supplemental material [file IAI.00010-17_zii999091993s1.pdf]

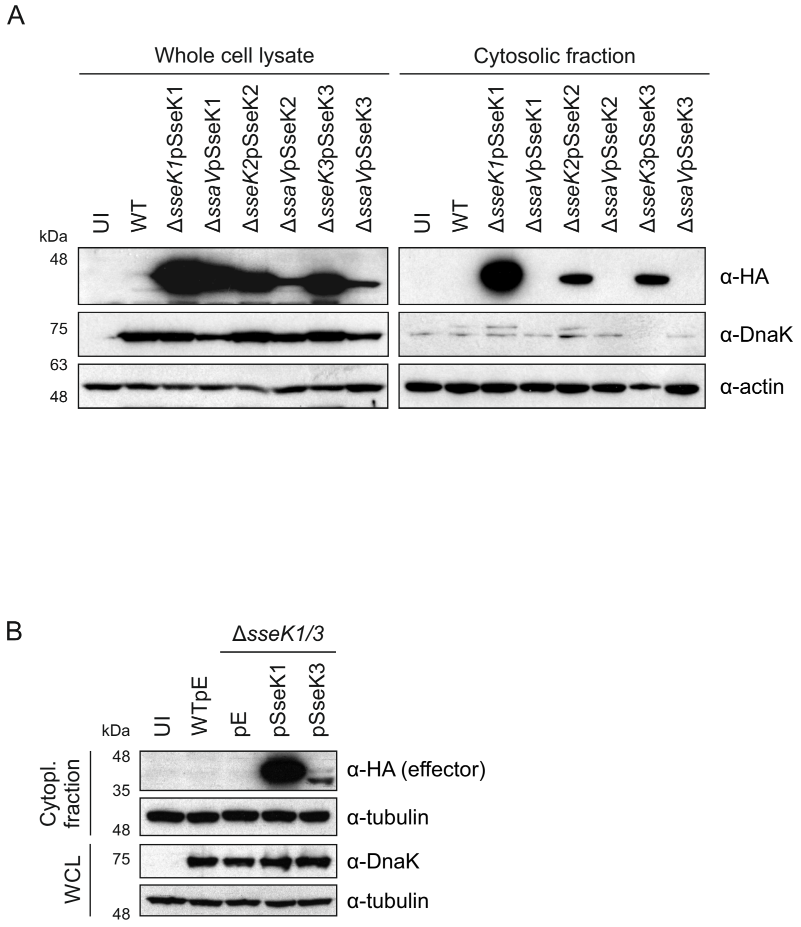


**Fig S1. SPI-2 T3SS dependent translocation of SseK effectors into macrophages**

(A-B) RAW264.7 macrophages were infected with the indicated *Salmonella* strains for 16 h. To compensate for the severe replication deficit Δ*ssaV* deletion mutant strains were used at 10x the MOI compared to WT *Salmonella*. Effector expression and translocation was analysed in whole cell lysates (WCL) and post-nuclear supernatants by SDS-PAGE and immuno-blotting of effectors (α-HA), *Salmonella* (α-DnaK), loading control (α-actin, α-tubulin). Data representative of two independent experiments. UI; uninfected.

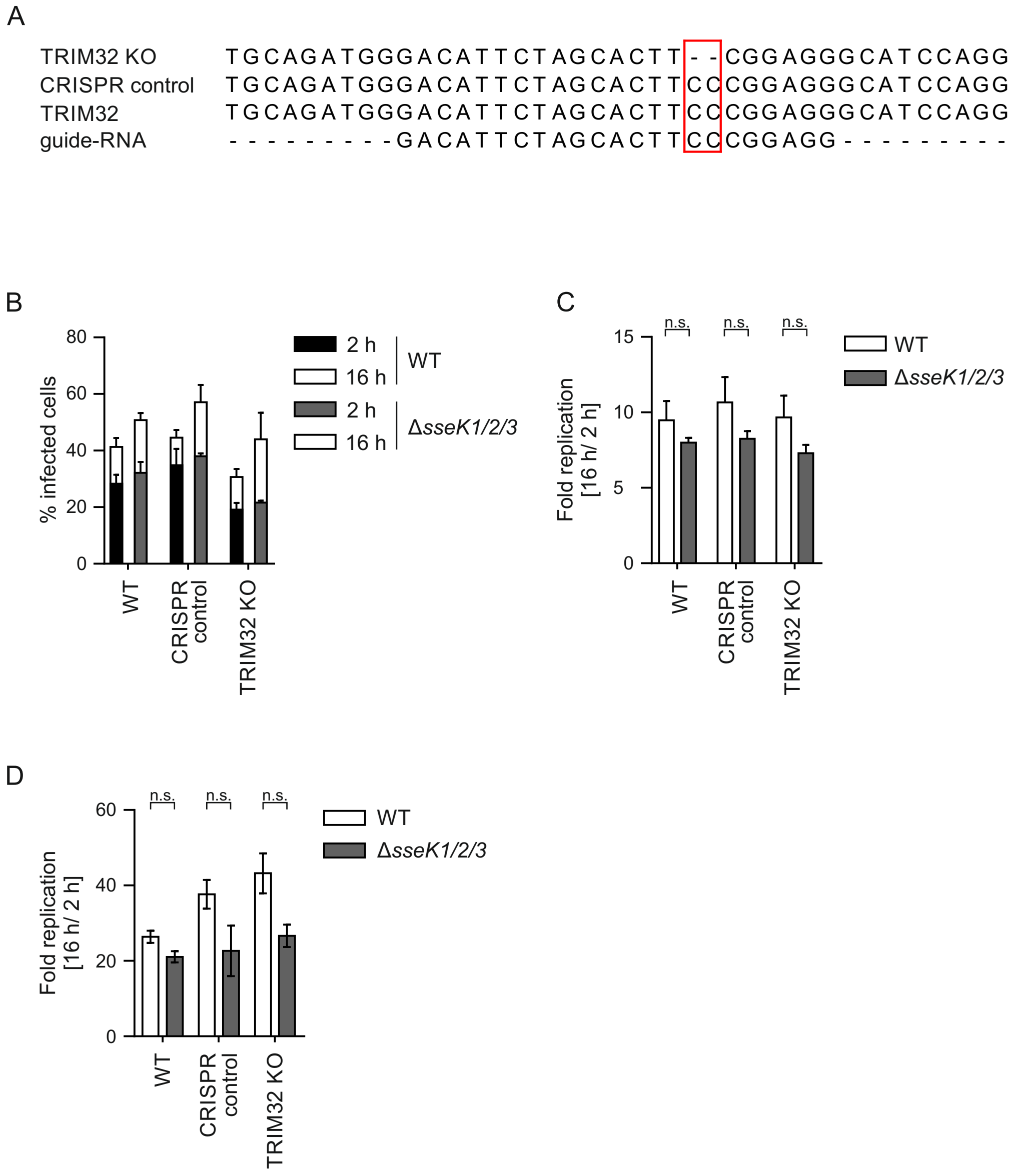


**Fig S2. Analysis of the TRIM32 knockout RAW264.7 macrophages**

(A) Sequence analysis of *Trim32* in WT and CRISPR generated TRIM32 knockout RAW264.7 macrophages. Alignment generated with Jalview. (B-C) TRIM32 knockout RAW264.7 macrophages were infected with the indicated GFP-expressing *Salmonella* strains for 2 h and 16 h. The infection rate (B) and fold geometric mean (C) were calculated after analysis by flow cytometry. The data represents the mean ± s.e.m. of three independent experiments. n.s. non significant. (D) TRIM32 deletion in macrophages does not affect *Salmonella* replication. Bacterial replication was analysed by gentamicin protection assay and calculated as fold colony-forming units (CFU) between 2 and 16 h.p.u. Values are the mean of three independent experiments ± s.e.m. Statistical analysis with Students *t*-test, differences not significant (n.s.).

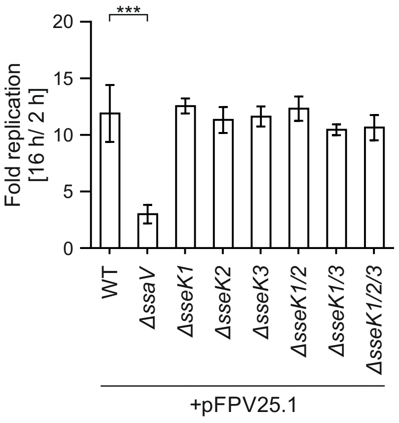


**Fig S3. SseK deletion strains do not have a replication defect in macrophages**

RAW264.7 macrophages were infected with the indicated GFP-expressing *Salmonella* strains for 2 h and 16 h. The GFP geometric mean was analysed by flow cytometry and the bacterial fold replication calculated. Data represents mean of three independent experiments ± s.e.m. *** $P < 0.001$.

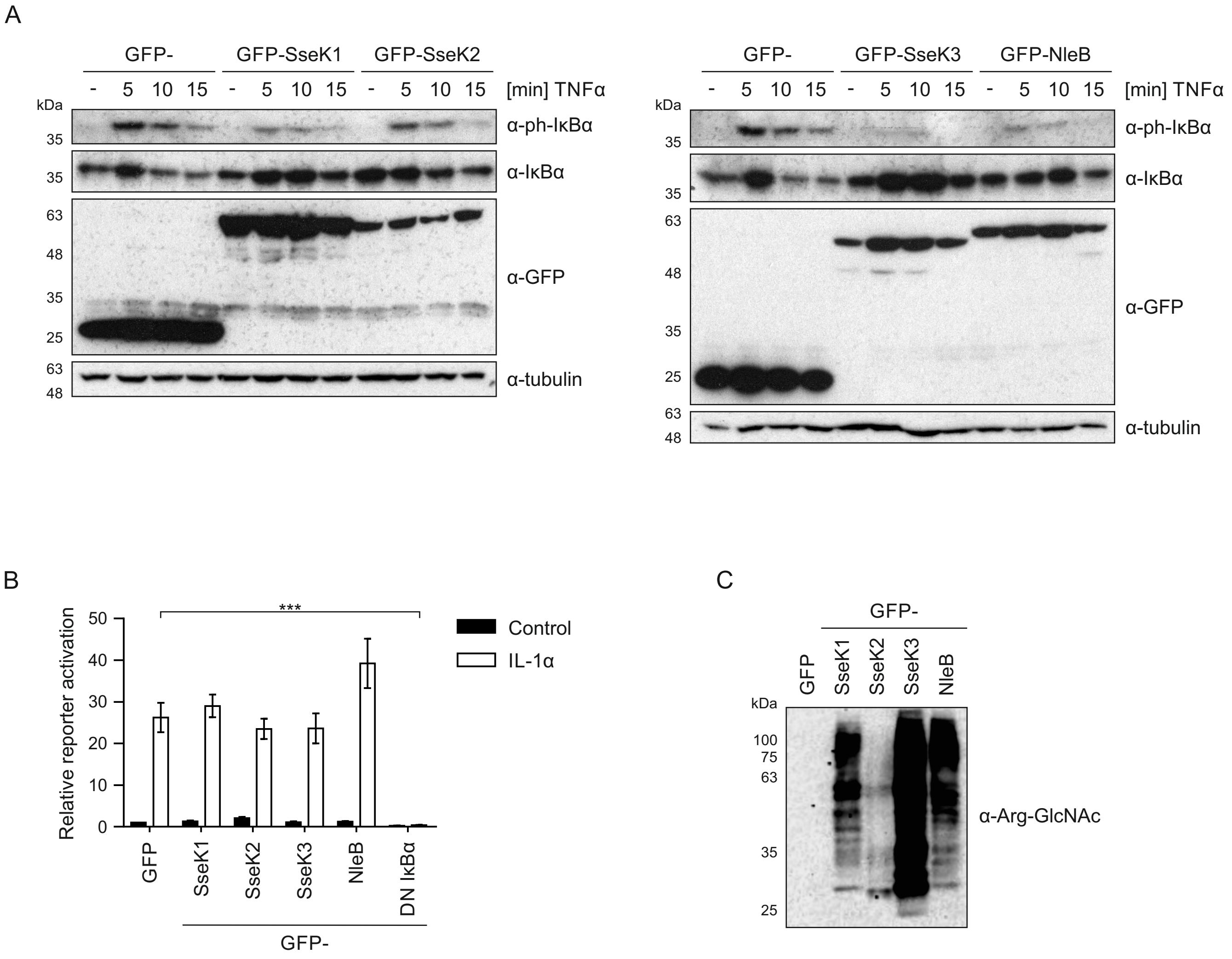


**Fig S4. *Salmonella* SseK effectors inhibit TNFα mediated IκBα phosphorylation but do not inhibit IL-1α mediated NF-κB signalling**

Inhibition of NF-κB signalling by GFP-SseK effectors following TNFα stimulation of transected 293ET cells. Cells were harvested after the indicated incubation with 50 ng/ml TNFα, lysed and analysed by SDS-PAGE and immuno-blotting to determine IκBα phosphorylation. Phospho IκBα (α-ph-IκBα), IκBα (α-IκBα), effectors (α-GFP), loading control (α-tubulin). (B) 293ET cells were co-transfected with an NF-κB -dependent luciferase reporter plasmid, pTK-Renilla luciferase plasmid and the indicated ptCMV-GFP-effector plasmids or dominant negative IκBα as a control. The NF-κB pathway was activated with 10 ng/ml IL-1α and luciferase activity measured in cell lysates after overnight stimulation. Results are presented as fold activation relative to unstimulated, GFP expressing control cells. These results were acquired at the same time as Fig. 3D and contain the same unstimulated data. Data shown are mean of five independent experiments ± s.e.m. *** $P < 0.001$. (C) SDS-PAGE and immuno-blotting of Arginine-GlcNAcylated proteins (α-Arg-GlcNAc) after expression of GFP-tagged effectors. This is a long exposure of Fig. 3C

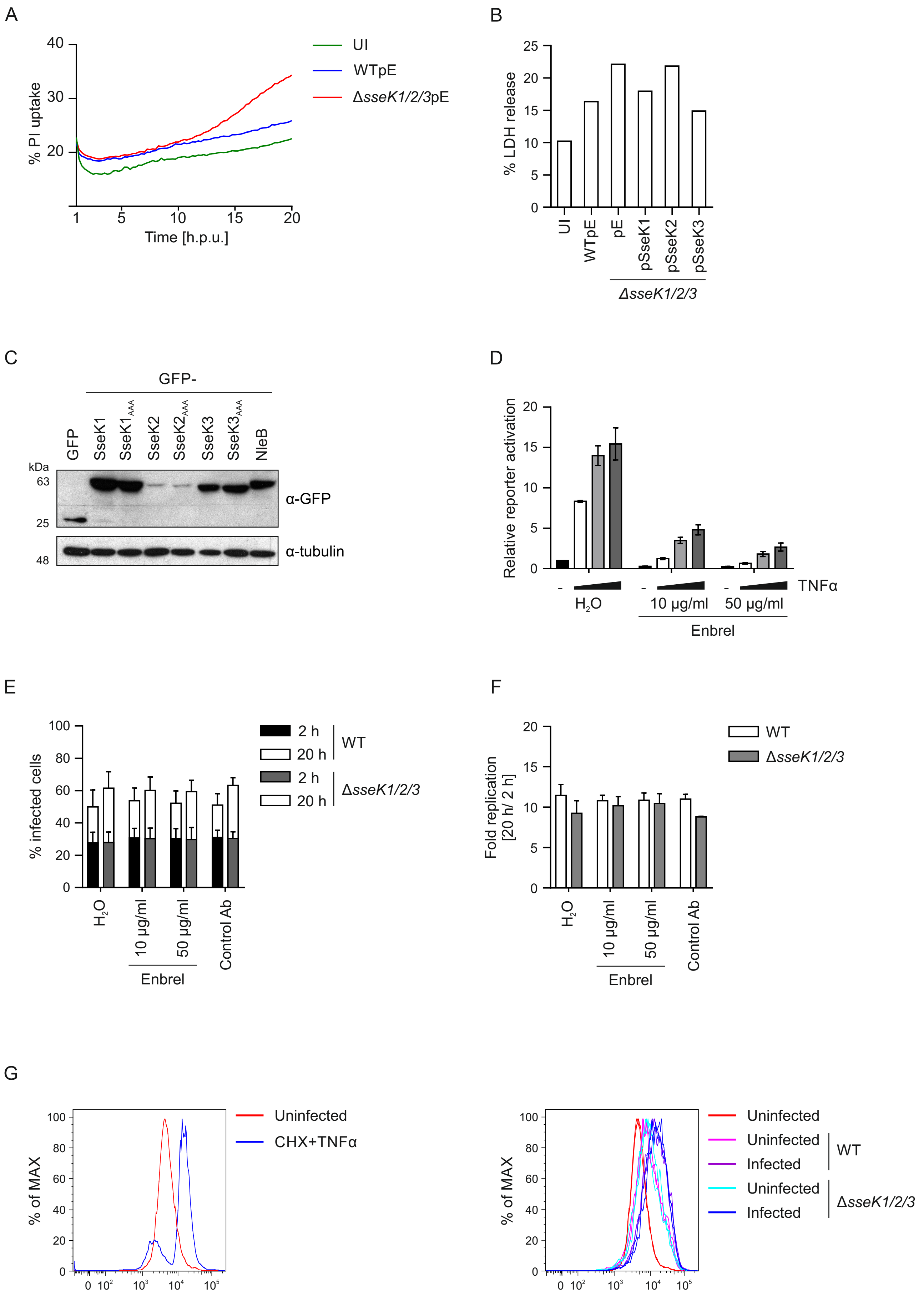

A
% PI uptake
Time [h.p.u.]
UI
WTpE
ΔsseK1/2/3pE
B
% LDH release
UI
WTpE
pE
pSseK1
pSseK2
pSseK3
ΔsseK1/2/3
C
GFP-
GFP
SseK1
SseK1AAA
SseK2
SseK2AAA
SseK3
SseK3AAA
NleB
kDa
63
25
48
α-GFP
α-tubulin
D
Relative reporter activation
TNFα
H2O
10 µg/ml
50 µg/ml
Enbrel
E
% infected cells
2 h
20 h
WT
ΔsseK1/2/3
H2O
10 µg/ml
50 µg/ml
Enbrel
Control Ab
F
Fold replication [20 h/ 2 h]
WT
ΔsseK1/2/3
H2O
10 µg/ml
50 µg/ml
Enbrel
Control Ab
G
% of MAX
Uninfected
CHX+TNFα
Uninfected
Uninfected
Infected
WT
Uninfected
Infected
ΔsseK1/2/3

**Fig S5. SseK effectors inhibit TNFα driven cell death**

(A) Representative experiment of Fig. 8A. RAW264.7 macrophages were infected with wild-type or mutant *Salmonella* strains and cell death assayed by propidium iodide (PI) uptake over time. (B) Representative experiment of Fig. 8B. LDH release from RAW264.7 macrophages infected for 20 h with the indicated *Salmonella* strains. Cell death was calculated relative to max. (C) Cell lysates from Fig. 8C were analysed by SDS-PAGE and immuno-blotting to determine effector protein expression levels during transfection LDH experiments. Effectors (α-GFP), loading control (α-tubulin). (D) The TNFα inhibitor Enbrel (Etanercept) inhibits TNFα driven NF-kB pathway activation in RAW264.7 macrophages. Cells were pre-incubated with Enbrel for 30 min and then stimulated with TNFα (10, 50, 100 ng/ml) for 16 h. Data is normalised to untreated water control cells and is the mean of three independent experiments ± s.e.m. (E-F) RAW264.7 macrophages were pre-treated with the indicated drugs for 30 min and then infected in the presence of the inhibitor for 2 and 20 h with the indicated GFP-expressing strains. The control antibody (Ab) was used at 50 μg/ml. The infection rate (E) and fold geometric mean (F) was calculated after analysis by flow cytometry. Data represents the mean of three independent experiments and differences are not significant. (G) Representative histograms of Fig. 8F. Caspase-3/7 activity was analysed using the SR-DEVD-FMK FLICA probe at 20 h.p.u. in infected RAW264.7 macrophages. Treatment with 50 μg/ml cyclohexamide and 50 ng/ml TNFα for 20 h was used as a positive control.

## Table S1

| ***S.* Typhimurium 12023 strains** | | |
|---|---|---|
| **Name** | **Description** | **Source or Reference** |
| wild-type | 12023 *S.* Typhimurium wild-type | NTCC |
| Δ*ssaV* | Δ*ssaV*::km | (1) |
| Δ*sseL* | Δ*sseL*::km | (2) |
| Δ*sseK1* | Δ*sseK1*::km | (3) |
| Δ*sseK2* | Δ*sseK2*::km | (3) |
| Δ*sseK3* | Δ*sseK3*::km | (3) |
| Δ*sseK1/2* | Δ*sseK1*/Δ*sseK2*::km | (3) |
| Δ*sseK1/3* | Δ*sseK1*/Δ*sseK3*::km | (3) |
| Δ*sseK1/2/3* | Δ*sseK1*/Δ*sseK2*/Δ*sseK3*::km | (3) |

## Table S2

| **Plasmids** | | |
|---|---|---|
| **Name** | **Description** | **Source or Reference** |
| pFPV25.1 | *rpsM::gfpmut3a* promoter fusion in pFPV | (4) |
| pWSK29-SseL-2HA | pWSK29 containing C-terminal 2HA-tagged SseL with 300 bp endogenous promoter ($Carb^R$) | (5) |
| pE | pWSK29 empty control plasmid ($Carb^R$) | (6) |
| pSseK1 | pWSK29 containing C-terminal 2HA-tagged SseK1 with 200 bp endogenous promoter ($Carb^R$) | This study |
| pSseK2 | pWSK29 containing C-terminal 2HA-tagged SseK2 with 200bp endogenous promoter ($Carb^R$) | This study |
| pSseK3 | pWSK29 containing C-terminal 2HA-tagged SseK3 with 1000 bp endogenous promoter ($Carb^R$) | This study |
| m3psinrevκB-*luc* | *luc* gene under control of NF-κB consensus promoter | F. Randow |
| m6pPAC-RLuc | Constitutively active Renilla luciferase for macrophage transduction | This study |
| pEGFP-N1 | GFP control plasmid | Clontech |
| ptCMV-GFP-SseK1 | SseK1 with N-terminal GFP-tag | This study |

| | | |
|---|---|---|
| ptCMV-GFP-SseK1$_{AAA}$ | SseK1 mutant DAD$_{223-225}$ mutated to AAA with N-terminal GFP-tag | This study |
| ptCMV-GFP-SseK2 | SseK2 with N-terminal GFP-tag | This study |
| ptCMV-GFP-SseK2$_{AAA}$ | SseK2 mutant DAD$_{239-241}$ mutated to AAA with N-terminal GFP-tag | This study |
| ptCMV-GFP-SseK3 | SseK3 with N-terminal GFP-tag | This study |
| ptCMV-GFP-SseK3$_{AAA}$ | SseK3 mutant DAD$_{226-228}$ mutated to AAA with N-terminal GFP-tag | This study |
| ptCMV-GFP-NleB | NleB with N-terminal GFP-tag | This study |
| ptCMV-GFP-DN IκBα | Dominant negative human IκBα (S32A/ S36A) with N-terminal GFP-tag | This study |
| pRLTK | Constitutively active Renilla luciferase | F. Randow |
| p4κB:Luc | *luc* gene under control of NF-κB consensus promoter | F. Randow |
| pEAKMMP-AU1-TLR4 | Human TLR4 for NF-κB pathway activation | F. Randow |
| m4pGFP-SseK1 | SseK1 with N-terminal GFP-tag | This study |
| m4pGFP-SseK3 | SseK3 with N-terminal GFP-tag | This study |
| m4pGFP-NleB | NleB with N-terminal GFP-tag | This study |
| m4pGFP-FADD | Murine FADD with N-terminal GFP-tag | This study |
| m4pGFP-TRADD | Murine TRADD with N-terminal GFP-tag | This study |
| m6pPAC-FLAG-GFP | GFP control plasmid | This study |
| m6pPAC-FLAG-FADD | Murine FADD with N-terminal FLAG-tag | This study |
| m6pPAC-FLAG-FADD$_{R117A}$ | Murine FADD with Arginine 117 mutated to Alanine with N-terminal FLAG-tag | This study |
| m6pPAC-FLAG-TRADD | Murine TRADD with N-terminal FLAG-tag | This study |
| m6pPAC-FLAG-TRADD$_{R233A}$ | Murine TRADD with Arginine 233 mutated to Alanine with N-terminal FLAG-tag | This study |
| m6pPAC-FLAG-TRIM32 | Murine TRIM32 with N-terminal FLAG-tag | This study |

## Table S3

| **Cloning primers** | | | |
|---|---|---|---|
| **Use** | | **Species** | **5’ to 3’ nucleotide sequence** |
| 200 bp-SseK1 | FW | *S.* Typhimurium 12023 | CGCGGGGAATTCAAATATGATGCCATTTCTGG |
| 200 bp-SseK1 | REV | *S.* Typhimurium 12023 | CGCGGGGGATCCCTGCACATGCCTCGCCCATG |
| 200 bp-SseK2 | FW | *S.* Typhimurium 12023 | CGCGGGGAATTCAATGGGCGCTTAGGTTTAGAG |
| 200 bp-SseK2 | REV | *S.* Typhimurium 12023 | CGCGGGGGATCCCCTCCAAGAACTGGCAGTTA |
| 1000 bp-SseK3 | FW | *S.* Typhimurium 12023 | CGCGGGGAATTCCACAGCAATTAATCTTCTGCCCG |
| 1000 bp-SseK3 | REV | *S.* Typhimurium 12023 | CGCGGGGGATCCTCTCCAGGAGCTGATAGTCAAAC |
| SseK1 | FW | *S.* Typhimurium 12023 | CGCGGGACATGTCA ATGATCCCACCATTAAATAG |
| SseK1 | REV | *S.* Typhimurium 12023 | CGCGGGGCGGCCGCCTACTGCACATGCCTCGCCCATG |
| SseK1$_{AAA}$ | FW | *S.* Typhimurium 12023 | ATAGTGGGTGTATATATCTTGCTGCTGCTATGATTATCACGGAAAAACT |
| SseK1$_{AAA}$ | REV | *S.* Typhimurium 12023 | AGTTTTTCCGTGATAATCATAGCAGCAGCAAGATATATACACCCACTAT |
| SseK2 | FW | *S.* Typhimurium 12023 | CGCGGGACATGTCAATGGCACGTTTTAATGCCGC |
| SseK2 | REV | *S.* Typhimurium 12023 | CGCGGGGCGGCCGCTTACCTCCAAGAACTGGCAG |
| SseK2$_{AAA}$ | FW | *S.* Typhimurium 12023 | GCGGTGGGTGCATATATCTTGCTGCTGCTATGTTACTTACTGATAAACT |
| SseK2$_{AAA}$ | REV | *S.* Typhimurium 12023 | AGTTTATCAGTAAGTAACATAGCAGCAGCAAGATATATGCACCCACCGC |
| SseK3 | FW | *S.* Typhimurium 12023 | CGCGGGCCATGGCAATGTTTTCTCGAGTCAGAGGTTTTC |
| SseK3 | REV | *S.* Typhimurium 12023 | CGCGGG GCGGCCGCTTATCTCCAGGAGCTGATAGTC |
| SseK3$_{AAA}$ | FW | *S.* Typhimurium 12023 | GGTGGCTGCATATATCTTGCTGCTGCTATGTTACTTACAGGTAAAC |
| SseK3$_{AAA}$ | REV | *S.* Typhimurium 12023 | GTTTACCTGTAAGTAACATAGCAGCAGCAAGATATATGCAGCCACC |
| NleB | FW | *E.coli* O157:H7 Sakai | CGCGGGACATGTCCATGTTATCTTCATTAAATGTC |
| NleB | REV | *E.coli* O157:H7 Sakai | CGCGGGGCGGCCGCTTACCATGAACTGCTGG |

| | | | |
|---|---|---|---|
| FADD | FW | *Mus musculus* | CGCGGGACATGTCCATGGACCCATTCCTGGTGCTG |
| FADD | REV | *Mus musculus* | CGCGGGGCGGCCGCTCAGGGTGTTTCTGAGGAAGAC |
| FADD$_{R117A}$ | FW | *Mus musculus* | CTGGAAAAGACTGGCCGCCGAGCTGAAGGTGTC |
| FADD$_{R117A}$ | REV | *Mus musculus* | GACACCTTCAGCTCGGCGGCCAGTCTTTTCCAG |
| TRADD | FW | *Mus musculus* | CGCGGGACATGTCCATGGCAGCCGGTCAGAATGG |
| TRADD | REV | *Mus musculus* | CGCGGGCGTCTCCGGCCGTTAGGCCAGGCCGCCATCCG |
| TRADD$_{R233A}$ | FW | *Mus musculus* | CTCAAGTGGCGCAGGGTGGGGGCCTCGCTGCAGCGTAACTGTCG |
| TRADD$_{R233A}$ | REV | *Mus musculus* | CGACAGTTACGCTGCAGCGAGGCCCCCACCCTGCGCCACTTGAG |
| Trim32 | FW | *Mus musculus* | CGCGGGACATGTCCATGGCTGCGGCTGCAGCAGCTTC |
| Trim32 | REV | *Mus musculus* | CGCGGGGCGGCCGCTTAAGGGGTGGAATATCTTCTCAG |
| DN IκBα | FW | *Homo sapiens* | CGCGGGACATGTCCATGTTCCAGGCGGCCGAGCGCCCCCAGGAGTGGGC |
| DN IκBα | REV | *Homo sapiens* | CGCGGGGCGGCCGCTCATAACGTCAGACGCTGGCCTCCAAACACACAGT |
| RLuc | FW | | CGCGGGCCATGGCAATGACTTCGAAAGTTTATGA |
| RLuc | REV | | CGCGGGGCGGCCGCTTATTGTTCATTTTTGAGAA |

## References


1. **Deiwick J**, **Nikolaus T**, **Shea JE**, **Holden DW**, **Hensel M**, **Deiwick RG**, **Gleeson C**. 1998. Mutations in Salmonella Pathogenicity Island 2 ( SPI2 ) Genes Affecting Transcription of SPI1 Genes and Resistance to Antimicrobial Agents. J Bacteriol **180**:4775–4780.
2. **Rytkönen A**, **Poh J**, **Garmendia J**, **Boyle C**, **Thompson A**, **Liu M**, **Freemont P**, **Hinton JCD**, **Holden DW**. 2007. SseL, a Salmonella deubiquitinase required for macrophage killing and virulence. Proc Natl Acad Sci U S A **104**:3502–3507.
3. **Figueira R**, **Watson KG**, **Holden DW**, **Helaine S**. 2013. Identification of Salmonella pathogenicity island-2 type III secretion system effectors involved in intramacrophage replication of S. enterica serovar typhimurium: Implications for rational vaccine design. MBio **4**:1–10.
4. **Valdivia RH**, **Falkow S**. 1996. Bacterial genetics by flow cytometry: rapid isolation of Salmonella typhimurium acid-inducible promoters by differential fluorescence induction. Mol Microbiol **22**:367–378.
5. **Pruneda JN**, **Durkin CH**, **Geurink PP**, **Ovaa H**, **Santhanam B**, **Holden DW**, **Correspondence DK**, **Komander D**. 2016. The Molecular Basis for Ubiquitin and Ubiquitin-like Specificities in Bacterial Effector Proteases. Mol Cell **63**:1–16.
6. **Wang RF**, **Kushner SR**. 1991. Construction of versatile low-copy-number vectors for cloning, sequencing and gene expression in Escherichia coli. Gene **100**:195–199.
